# Synthesis and Biological Evaluation of New Quinazolin-4(3H)-One–Coumarin Hybrids Designed as Anticancer and Antibacterial Agents

**DOI:** 10.3390/ijms27104485

**Published:** 2026-05-16

**Authors:** Maria P. Paramonova, Mikhail S. Novikov, Vera A. Sokhraneva, Iulia S. Zhivotova, Vasiliy A. Kezin, Martin A. Zenov, Irina Yu. Petrushanko, Olga N. Novikova, Andrey V. Gorshenin, Yulia I. Velikorodnaya, Elena B. Isakova, Andrey E. Shchekotikhin, Sergey N. Kochetkov, Elena S. Matyugina, Anastasia L. Khandazhinskaya

**Affiliations:** 1 Department of Pharmaceutical and Toxicological Chemistry, Volgograd State Medical University, Volgograd 400131, Russia; mp_paramonova@mail.ru (M.P.P.); ms_novikov1@mail.ru (M.S.N.); velikorodnaya@rihtop.ru (Y.I.V.); 2 Engelhardt Institute of Molecular Biology, Russian Academy of Sciences, Moscow 119991, Russia; sokhraneva.v@mail.ru (V.A.S.); zhivotovajulia51@gmail.com (I.S.Z.); vassileves58@yandex.ru (V.A.K.); martin.zenov@yandex.ru (M.A.Z.); irina-pva@mail.ru (I.Y.P.); snk1952@gmail.com (S.N.K.); matyugina@gmail.com (E.S.M.); 3 Moscow Center for Advanced Studies, Moscow 123592, Russia; 4 Laboratory of Immunology, Federal State Unitary Enterprise “Research Institute of Hygiene, Toxicology, and Occupational Pathology” of the Federal Medical and Biological Agency of the Russian Federation, Volgograd 400048, Russia; novikova@rihtop.ru (O.N.N.); gorshenin@rihtop.ru (A.V.G.); 5 Laboratory of Pharmacology and Chemotherapy, Gause Institute of New Antibiotics, Moscow 119021, Russia; ebisakova@yandex.ru; 6 Laboratory of Chemical Transformation Antibiotic, Gause Institute of New Antibiotics, Moscow 119021, Russia; shchekotikhin@mail.ru

**Keywords:** quinazolines, coumarins, cytotoxicity, leukemia, neuroblastoma, antibacterial activity

## Abstract

Quinazolinone derivatives are well-known anticancer agents; anticancer properties are also part of the broad spectrum of biological activity of coumarins. Conjugates containing quinazolin-4(3H)-one and coumarin fragments linked by polymethylene bridges of varying lengths were designed to improve properties of both parental compounds and create new anticancer or antibacterial agents. 3-{3-[(4-Methyl-2-oxo-2H-chromen-7-yl)oxy]propyl}quinazolin-4(3H)-one was synthesized as the base compound. It demonstrated moderate cytotoxicity against leukemia (K562 and HL60) and neuroblastoma (SH-SY5Y) cells in vitro, combined with relatively low acute, subacute, and chronic toxicity in vivo. Conjugates with various substituents and linkers were then synthesized to evaluate the structure–activity relationship. A study of the synthesized compounds on cell cultures showed that the introduction of a methyl substituent into the benzene ring of the coumarin fragment led to both an increase in cytotoxicity and expansion of its spectrum of action. Testing of the hybrids against Gram-positive and Gram-negative bacteria revealed that the introduction of halogens into the quinazoline fragment in the compounds or the elongation of the linker led to the emergence of pronounced antibacterial properties, which were most clearly manifested against *Acinetobacter baumanii*. The possibility of directing activity of quinazoline-4(3H)-one–coumarin hybrids by varying the substituents and the length of the linker was shown.

## 1. Introduction

Currently, cancer is the second leading cause of death worldwide after cardiovascular diseases. The most common types are lung, breast, colon, cervical, and prostate cancer. Despite advances in diagnosis and treatment, mortality from malignant neoplasms continues to rise [1]. Cancer treatment methods include surgery, radiation therapy, immunotherapy, and chemotherapy. Immunotherapy is primarily used for hematological malignancies and metastatic cancer, while radiation therapy and surgery are used primarily for localized tumors. Chemotherapy remains the most widely used and effective systemic treatment [1].

**Figure 1:**
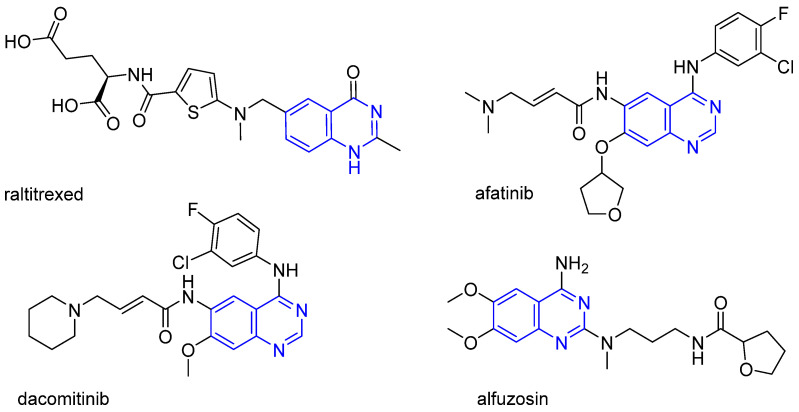
Structures of some quinazoline/quinazolinone derivatives approved as anticancer drugs. Quinazoline fragment colored in blue.

Quinazolines and quinazolinones are nitrogen-containing heterocyclic compounds with significant biological activity and therapeutic potential. Their chemical versatility, ease of synthesis, and ability to undergo structural modification make them attractive targets for research in medicinal chemistry. Several clinically approved drugs have been developed based on the quinazoline/quinazolinone structure, exhibiting a variety of pharmacological effects, including antibacterial, antituberculosis, antitumor, anticonvulsant, anti-inflammatory, and antiviral activity [2,3,4,5,6]. In oncology, quinazoline/quinazolinone derivatives such as raltitrexed (used in colorectal cancer), afatinib and dacomitinib (used in metastatic non-small-cell lung cancer), and alfuzosin (used in benign prostatic hyperplasia) are well-known chemotherapeutic agents [7,8,9,10] (Figure 1). EGFR (epidermal growth factor receptor) and VEGFR (vascular endothelial growth factor receptor) are the primary targets of many quinazoline derivatives, showing strong inhibition at nanomolar IC_50_ levels through hydrogen bonding and hydrophobic interactions within ATP-binding pockets. Additionally, quinazoline derivatives have shown notable cytotoxic and apoptosis-inducing effects acting on BRAF (B-rapidly accelerated fibrosarcoma), HER2 (human epidermal growth factor receptor 2), PARP-1 (Poly(ADP-ribose) polymerase-1), COX-2 (Cyclooxygenase-2), and PI3K/Akt/mTOR (Phosphoinositide 3-kinase/ Protein kinase B/Mammalian target of rapamycin) pathways [11].

Quinazolin-4(3H)-one derivatives exhibit potent antitumor [12] and antibacterial activity [13]. For example, El-Badry et al. synthesized a series of 2,3-disubstituted 4(3H)-quinazolin-4-ones and evaluated their antimicrobial and anticancer properties [14]. Although their antimicrobial activity against Gram-positive and Gram-negative bacteria and pathogenic fungi was limited compared to standard drugs, some compounds demonstrated significant cytotoxicity against human tumor cell lines, including melanoma, ovarian, kidney, prostate, breast, and colon cancer. Some derivatives demonstrated superior activity compared to 5-fluorouracil.

Despite their therapeutic potential, quinazoline-based drugs have side effects such as allergic reactions, withdrawal syndrome, overdose toxicity, and drug interactions. For example, raltitrexed can cause bone marrow suppression, gastrointestinal dysfunction, and oral mycoses [15]. Toxicology studies have shown LD_50_ (Lethal dose causing death in 50% of experimental animals) values of 875 mg/kg in mice and 1249 mg/kg in rats, with doses ≥ 750 mg/kg causing death due to systemic toxicity [16]. Chronic administration studies in rats and dogs have revealed cytotoxicity targeting proliferative tissues, including the gastrointestinal tract, bone marrow, thymus, and reproductive organs. Raltitrexed also impaired male fertility, with reversible effects upon discontinuation, and caused embryolethality, fetal malformations, and chromosomal damage at cytotoxic doses [16].

Similarly, administration of afatinib during pregnancy in animal models resulted in increased postimplantation mortality, fetal growth retardation, and developmental abnormalities at doses exceeding the equivalent human dose [17]. These findings highlight the broad spectrum of toxic effects associated with quinazoline derivatives affecting vital organ systems such as the gastrointestinal, hepatobiliary, and hematopoietic systems [15,16].

Nevertheless, given the growing number of new compounds with antitumor activity, diverse molecular targets, and mechanisms of action, the development of new quinazoline-based drugs remains a promising direction in cancer chemotherapy. Comprehensive toxicological evaluation is crucial for establishing safe dosing regimens for future clinical applications.

**Figure 2:**
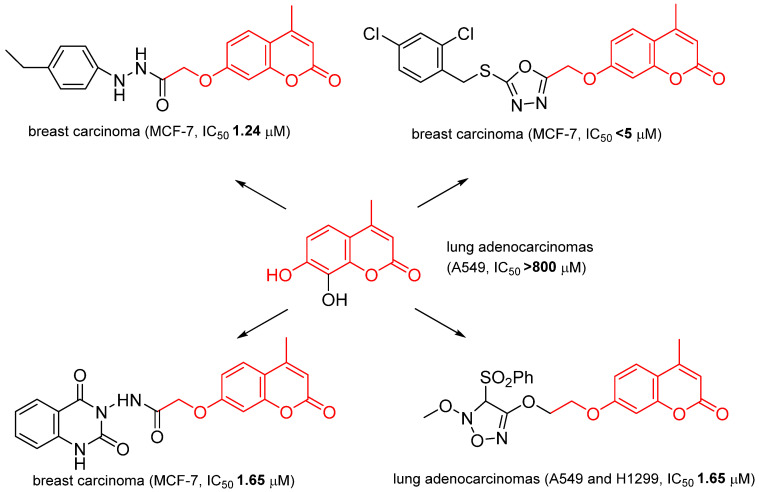
Structures of some 4-methyl-coumarin derivatives with anticancer activity. Coumarin fragment colored in red.

Coumarins are components of various natural compounds and therefore exhibit a broad spectrum of biological activity. They act as growth inhibitors and exhibit protective properties against plant diseases caused by pathogens. At the same time, due to their anticoagulant activity, coumarins (dicumarin) can have a toxic effect on mammals, causing bleeding. Furthermore, coumarins have diverse pharmacological activity, exhibiting antispasmodic, cardiovascular, photosensitizing, and antitumor effects [18,19,20]. Among the coumarin derivatives, unlike the quinazoline derivatives described above, there are no approved anticancer drugs. However, many derivatives have shown activity against various types of cancer [21]. The mechanisms of anticancer action include induction of apoptosis (caspase activation and BAX (Bcl-2-associated X protein)/BCL-2 (B-cell lymphoma 2) balance), modulation of PI3K/Akt/mTOR signaling, inhibition of angiogenesis (VEGFR-2), interference with estrogen biosynthesis (aromatase/ estrogen receptor (ER) axis), chaperone targeting (Heat shock protein 90, Hsp90), etc. [21]. Among other compounds, various 4-methylcoumarin derivatives have been studied as anticancer agents. Notably, 7,8-diacetoxy-4-methylcoumarin did not exhibit significant activity, but the introduction of various heteroaromatic substituents, including quinazolin-2,4-dione, at the 7-position resulted in pronounced anticancer properties [21] (Figure 2).

**Figure 3:**
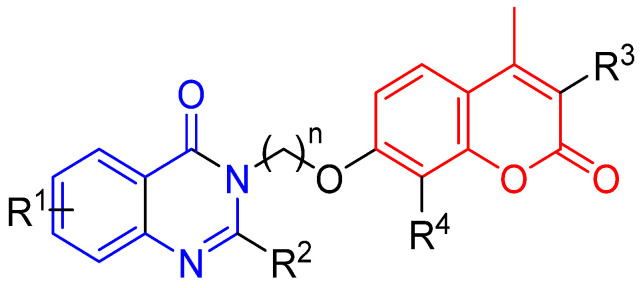
New chimeric molecules containing quinazolin-4(3H)-one and coumarin fragments.

So, combining two pharmacophoric fragments, quinazolin-4(3H)-one and coumarin, in one molecule appears promising. In this study, the in vitro cytotoxicity and acute, subchronic, and chronic toxicity in vivo of a base compound containing quinazolin-4(3H)-one and coumarin fragments (Figure 3) was assessed. In order to optimize the structure, new chimeric molecules in which the quinazolin-4(3H)-one and coumarin moieties containing various substituents and connected by linkers of varying lengths were designed and synthesized. The antiproliferative activity of the synthesized compounds against leukemia (K562 and HL60) and neuroblastoma (SH-SY5Y) cells was evaluated. Additionally antibacterial properties of quinazolin-4(3H)-one–coumarin hybrids were tested against Gram-positive and Gram-negative bacteria since chemotherapy can induce immune suppression and makes cancer patients vulnerable to hospital-acquired infections.

## 2. Results and Discussion

**Scheme 1:**
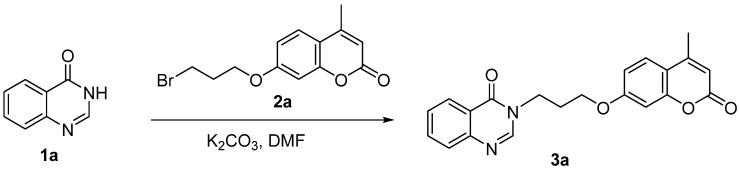
Synthesis of 3-{3-[(4-Methyl-2-oxo-2H-chromen-7-yl)oxy]propyl}quinazolin-4(3H)-one (**3a**).

Previously, a series of uracil–coumarin conjugates that exhibited high activity against human cytomegaloviruses in vitro were synthesized [22]. This work presents the synthesis of new compounds containing quinazolin-4(3H)-one and coumarin fragments linked by polymethylene bridges of varying lengths. In the first step, 3-{3-[(4-methyl-2-oxo-2H-chromen-7-yl)oxy]propyl}quinazolin-4(3H)-one (**3a**) was synthesized from quinazolin-4(3H)-one (**1a**) and 7-(3-bromopropyloxy)-4-methyl-2H-chromene (**2a**) by heating in the presence of potassium carbonate in DMF (Scheme 1); the yield was 82%.

Evaluation of the antiproliferative potency of conjugate **3a** was performed on SH-SY5Y neuroblastoma cell lines, K-562 lymphoblastic cells and HL-60 promyeloblastic cells, Jurkat T-cell leukemia cells, HeLa cervical tumor cells, and HepG2 hepatocellular carcinoma cell line, as well as on U87 and U251 glioblastoma cell lines. Results of screening showed that derivative **3a** inhibited growth of SH-SY5Y and Jurkat cells in micromolar concentrations (IC_50_ 27µM for both cell lines) without reliable effect to other cells.

**Figure 4:**
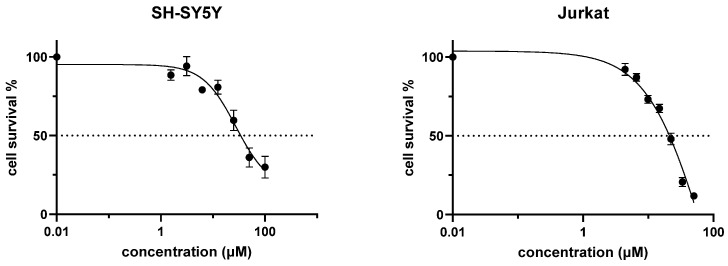
Cell viability after treatment with compound **3a**.

Representative viability curves (Figure 4) demonstrate a concentration-dependent decrease in the viability of SH-SY5Y and Jurkat cells upon exposure to compound **3a**. These results indicate that compound **3a** exhibits antiproliferative activity against both neuroblastoma and leukemia cells, the IC_50_ values of which were determined based on the plotted curves.

As a result of studies of the acute toxicity of compound **3a**, it was established that when administered *per os* at the maximum possible dose of 250 mg/kg, there were no clinical manifestations of intoxication or death of the experimental animals at any time during the observation period.

At autopsy, after the end of the 14-day observation period, no macro- or microscopic changes in the internal organs were observed in animals that were administered compound **3a** at a dose of 250 mg/kg. Therefore, the tested dose was assessed as LD_0_.

However, subchronic two-week administration of compound **3a** to nonlinear mice at a daily dose of 100 mg/kg resulted in minor changes in the studied homeostasis parameters, which disappeared 14 days after the end of treatment (Table 1).

**Figure 5:**
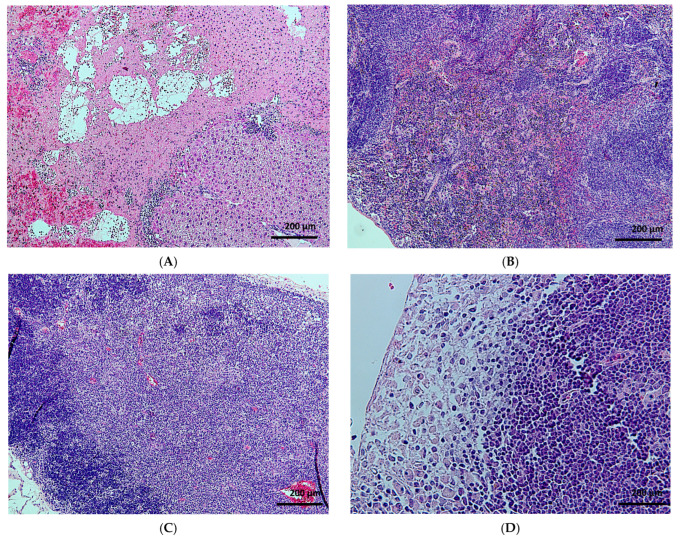
Hematoxylin and eosin staining. Magnification: 100 (**A**–**C**) and 200 (**D**). Fragments of the liver (**A**), spleen (**B**), thymus (**C**), and small intestinal lymphoid follicle (**D**) of female experimental mice 14 days after subchronic administration of compound **3a** at a dose of 100 mg/kg.

The results of the pathomorphological examination of the organs and tissues of the experimental mice 14 days after subchronic administration of compound **3a** at a dose of 100 mg/kg showed that the tested compound can negatively affect the hepatobiliary and immune systems of female individuals. Thus, in the liver parenchyma of one experimental female mouse, large foci of hemorrhagic necrosis were recorded, associated with leukocyte infiltration and disruption of the protein–water–electrolyte metabolism of hepatocytes, up to their hydropic degeneration and death (Figure 5A). Also noted was one case of a sharp decrease in the number of follicles in the spleen (Figure 5B), an increase in the area of the medulla in the thymus (Figure 5C), and the death of immunocompetent cells of the lymphoid follicle of the small intestine (Figure 5D).

During the pathomorphological examination of organs and tissues of male mice during this period, no significant differences from the control were noted. It is known that sexual sensitivity to the effects of toxicants is determined by differences in the rate of their biotransformation in liver cells, as well as by the biological specificity of the effects of male and female sex hormones.

At the end of the recovery period following subchronic administration of compound **3a** at a dose of 100 mg/kg, the microscopic structure of organs and tissues of experimental mice of both sexes corresponded to normal histological criteria.

With repeated (within 2 months) administration of this compound in various doses, a number of statistically significant changes in physiological, hematological, and biochemical parameters were also observed in animals (Table 2).

**Figure 6:**
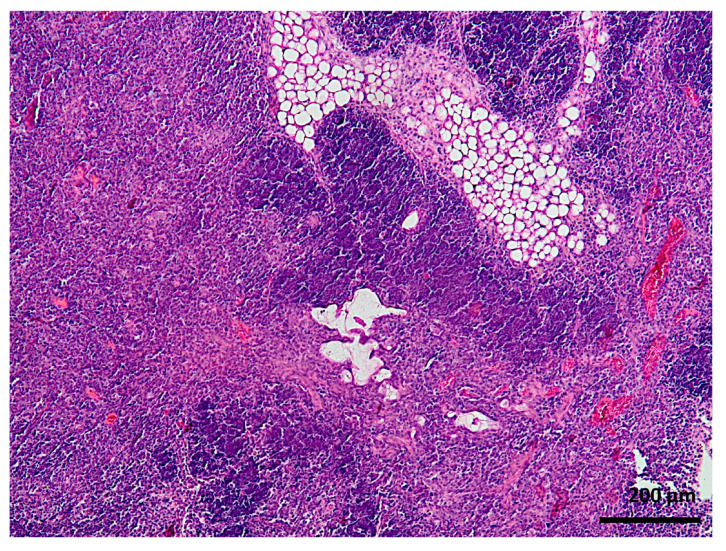
Thymus fragment from a female experimental mouse 1 month after intragastric administration of compound **3a** at a dose of 4 mg/kg. Hematoxylin and eosin staining. Magnification: 100.

**Figure 7:**
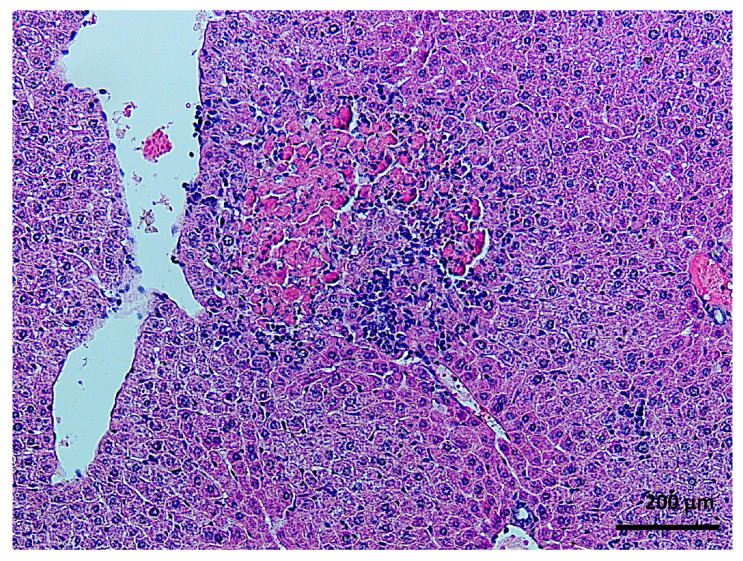
Liver tissue fragment from a female experimental mouse 1 month after intragastric administration of compound **3a** at a dose of 20 mg/kg. Hematoxylin and eosin staining. Magnification: 200.

A pathological study revealed that chronic exposure to a low dose of compound **3a** (4 mg/kg) can negatively impact the immune system of female mice. For example, one mouse showed histological signs of thymus involution, characterized by a significant predominance of medulla over cortex and an overgrowth of connective and adipose tissue in the organ parenchyma (Figure 6). Administration of a higher dose (20 mg/kg) induced focal death of hepatocytes in one female mouse, which was accompanied by the attraction of inflammatory elements to the damaged area (Figure 7).

**Figure 8:**
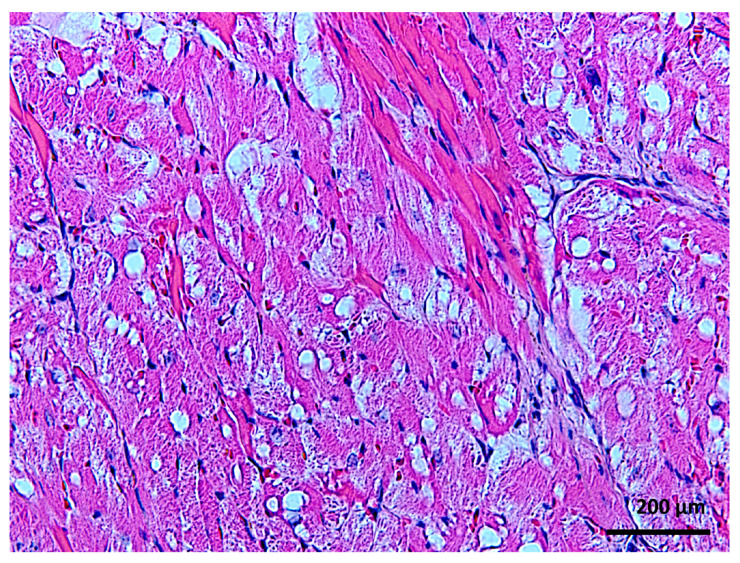
Myocardial fragment from a male experimental mouse 1 month after chronic administration of compound **3a** at a dose of 4 mg/kg. Hematoxylin and eosin staining. Magnification: 400.

Microscopic evaluation of the organs and tissues of male experimental mice revealed that the most pronounced cellular changes following administration of 4 mg/kg **3a** were observed in the myocardium of two mice. The cytoplasm of individual cardiomyocytes became more homogeneous and oxyphilic. Numerous vacuoles were detected in the cytosol of other more numerous myocardial cells, often disrupting the ordered arrangement of myofibrils, while the cardiomyocytes themselves increased in diameter and acquired a more rounded shape (Figure 8).

**Figure 9:**
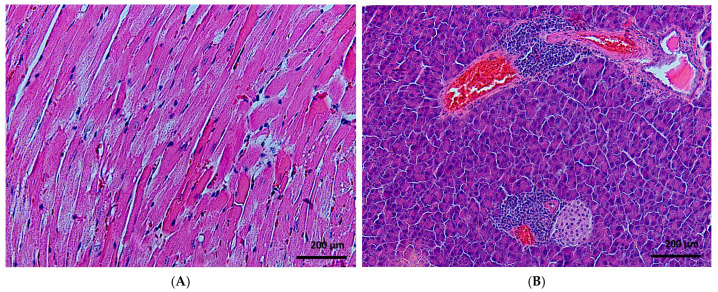
Fragment of the myocardium (**A**) and pancreas (**B**) of male experimental mice 1 month after chronic administration of compound **3a** at a dose of 20 mg/kg. Hematoxylin and eosin staining. Magnification: 400 (**A**) and 200 (**B**).

Similar changes in cardiac structure were observed in one male experimental mouse receiving 20 mg/kg **3a** for 1 month (Figure 9A). Furthermore, large perivascular infiltrates formed in the pancreas of one animal following administration of a higher dose of **3a** (Figure 9B).

**Figure 10:**
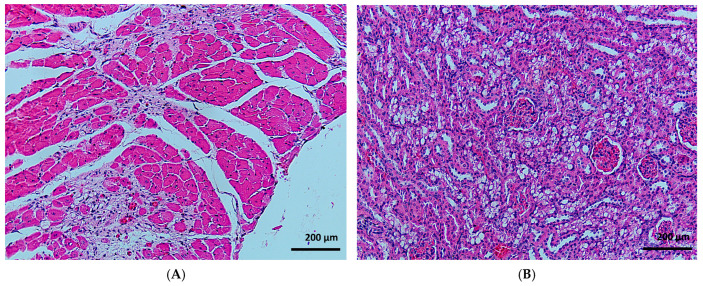
Myocardial (**A**) and renal (**B**) fragments from male experimental mice 2 months after chronic administration of compound **3a** at a dose of 4 mg/kg. Hematoxylin and eosin staining. Magnification: 200.

Microscopic studies of the organs and tissues of male mice after two months of the experiment revealed that continuous intragastric administration of 4 mg/kg of **3a** induced fibrogenesis in the cardiac muscle of one experimental animal, leading to the proliferation of connective tissue in the myocardium (Figure 10A). In another male, the negative effect of prolonged exposure to **3a** was detected in the epithelial cells of the renal convoluted tubules, where large optically transparent vacuoles formed (Figure 10B).

**Figure 11:**
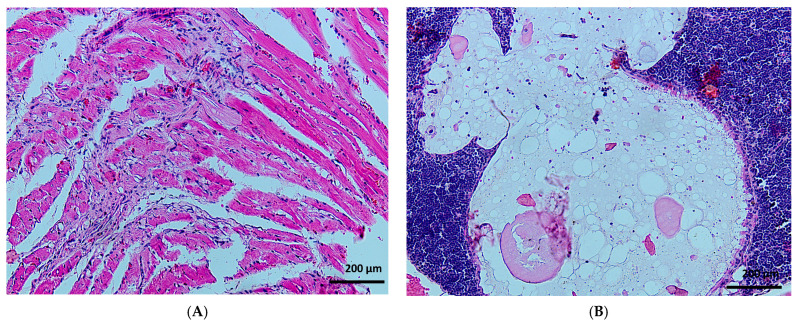
Myocardial fragment (**A**) and lymph node (**B**) from male experimental mice 2 months after chronic administration of compound **3a** at a dose of 20 mg/kg. Hematoxylin and eosin staining. Magnification: 200.

Following administration of a higher dose of compound **3a** for 2 months, pathological changes in the myocardium, manifested by focal interstitial edema and fibrosis, were also observed in one male (Figure 11A). Another male developed a large cystic cavity filled with foamy material in the stroma of a mediastinal lymph node (Figure 11B).

**Figure 12:**
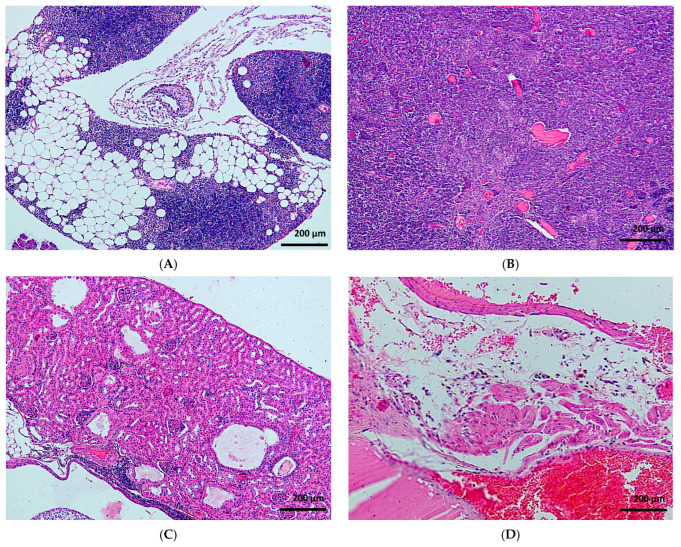
Fragments of the thymus (**A**,**B**), kidney (**C**), and myocardium (**D**) of female experimental mice 2 months after chronic administration of compound **3a** at a dose of 4 mg/kg. Hematoxylin and eosin staining. Magnification: 100.

Pathological examination of the organs and tissues of experimental female mice after chronic intragastric administration of **3a** at a dose of 4 mg/kg for 2 months revealed that the primary adverse reaction to the substance occurred in the thymus. One experimental mouse exhibited active replacement of glandular tissue by adipose tissue, which is not typically observed in rodents (Figure 12A), while another individual had numerous cystic lesions filled with oxyphilic fluid in the medulla (Figure 12B). Also in this experimental group, one case of large cystic cavities filled with transudate was recorded in the renal cortex (Figure 12C), and one case of severe interstitial edema of the right ventricular wall, leading to connective tissue destruction and the death of some cardiomyocytes (Figure 12D). At the same time, chronic administration of **3a** at a dose of 20 mg/kg for 2 months did not lead to gross changes at the cellular level in the studied organs of female experimental mice; no significant differences from the control were noted.

After the recovery period, a pathological examination revealed that administration of compound **3a** for two months did not result in profound structural changes in organs and tissues. Most of the observed morphological changes were reversible, and after discontinuation of the test compound, the histoarchitecture of the examined organs and tissues in the mice met normal histological criteria.

**Figure 13:**
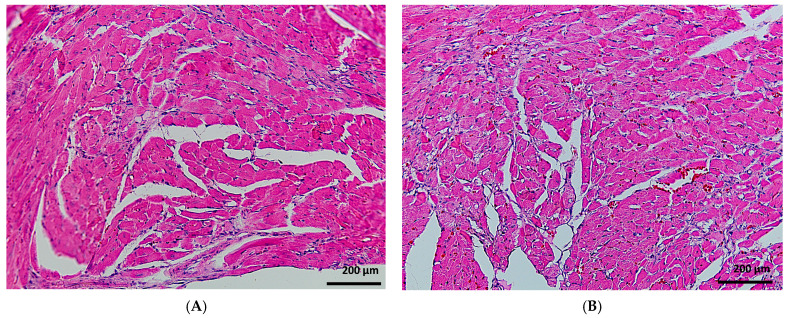
Myocardial fragments from male experimental mice at the end of the recovery period following chronic administration of compound **3a** at a dose of 4 mg/kg (**A**) and 20 mg/kg (**B**). Hematoxylin and eosin staining. Magnification: 200.

At the same time, in male mice (two individuals from each experimental group), the consequences of the negative impact of the substance on the myocardium were revealed, as evidenced by the proliferation of connective tissue layers between cardiomyocytes in the area of damage to the heart muscle (Figure 13).

Acute toxicity studies of compound **3a** in laboratory animals demonstrated a relatively low toxicity profile. The maximum dose tested (250 mg/kg) did not cause any toxic effects and was designated as LD_0_. Comparison of these data with the literature data on the toxicity of raltitrexed [15,16] suggests that the median lethal dose (LD_50_) of compound **3a** in mice may be in the range of 750–875 mg/kg and higher. To accurately determine the acute toxicity parameters of compound **3a**, the development of an innovative formulation that would increase its bioavailability and allow administration at doses higher than those studied will likely be necessary.

The results of subchronic and chronic experiments showed that with prolonged monotonous introduction into the body, the substance is capable of causing disturbances in various vital parameters.

At the same time, a short-term administration of a higher dose (14 days, 100 mg/kg) led to the appearance of a minor spectrum of changes in the studied parameters and pathomorphological shifts (only in females), which were reversible.

Meanwhile, prolonged exposure (2 months) to lower doses of **3a** (4 mg/kg and 20 mg/kg) was accompanied by shifts in a significant number of parameters in both experimental groups throughout the entire observation period. Thus, during the chronic experiment mice exhibited disturbances, clearly indicating a decrease in endurance and motor activity, as well as indirectly confirming stress on the hematopoietic system, liver, and heart.

Changes in physiological, hematological, and biochemical parameters were accompanied by tissue modifications detected by histological examination. The substance at both doses tested caused histoarchitectonic changes in a number of internal organs, with the thymus most frequently affected in females, and the myocardium in males. In some animals, the liver, kidneys, and pancreas served as target organs. Similar morphological changes were also detected in female mice on day 14 in a subacute experiment.

After a month-long recovery period following the chronic experiment, all previously altered homeostasis parameters returned to normal. However, during this period, a decrease in platelet count and thrombocrit was observed in both experimental groups, along with a decrease in serum urea concentrations in animals administered the substance at a dose of 20 mg/kg. Furthermore, according to histological examination results, the detected myocardial damage resulted in the development of focal cardiac fibrosis in some male mice, suggesting that substance **3a** has a cardiotoxic effect across the tested dose range.

It should be noted that both selected doses of the substance were effective and approximately equal in the depth and range of abnormalities induced with a slight predominance of overall cellular disturbances in mice from the first group (4 mg/kg). Furthermore, the results of histological studies conducted during subchronic and chronic experiments suggest that females are somewhat more sensitive to the effects of **3a** than males.

To summarize the above, it can be concluded that the presence of cytotoxicity in compound **3a** toward SH-SY5Y neuroblastoma cells and Jurkat T-cell leukemia in combination with a relatively low acute, subchronic, and chronic toxicity profile confirms the feasibility of its further study as a base compound for the development of new antitumor agents.

**Scheme 2:**
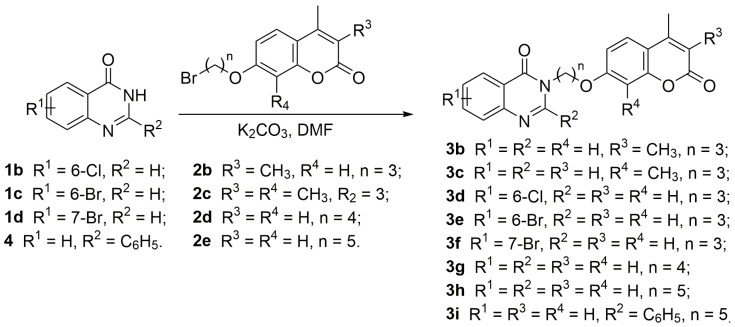
Synthesis of target compounds **3b–3i**.

To study the structure–activity relationships, new quinazolin-4(3H)-one–coumarin conjugates were designed, in which the quinazolin-4(3H)-one and coumarin fragments contain various substituents and are joined by linkers of different lengths. Compounds **3b**–**3i** were prepared similarly to the base conjugate, **3a**. The synthesis consisted of treating the starting quinazolin-4(3H)-ones **1b**–**1d** or 2-phenylquinazolin-4(3-H)-one (**4**) with an equimolar amount of 7-(ω-bromoalkoxy)-4-methyl-2H-chromenes **2b**–**2e** [22,23,24,25] in a DMF solution and in the presence of potassium carbonate. As a result, 3-{ω-[(4-methyl-2-oxo-2H-chromen-7-yl)oxy]alkyl}quinazolin-4(3H)-ones **3b**–**3i** were formed with 62–86% yield (see Scheme 2).

**Figure 14:**
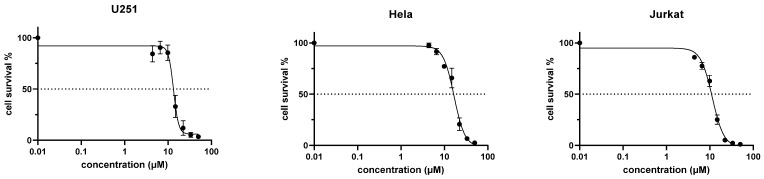
Representative viability curves demonstrate a concentration-dependent decrease in the viability of U251, Hela, and Jurkat cells upon exposure to compound **3c**.

Screening of the antiproliferative activity of synthesized derivatives was performed on the SH-SY5Y neuroblastoma cell line, K-562 lymphoblastic cells and HL-60 promyeloblastic cells, Jurkat T-cell leukemia cells, HeLa cervical tumor cells, HepG2 hepatocellular carcinoma cell line, and U87 and U251 glioblastoma cell lines (Table 3). 2′-Deoxy-5-fluorouridine (FUDR) was used as positive control. As a result of cytotoxicity assessment on the above cell lines, compound **3c** exhibited the highest activity (IC_50_ 11.0–47.0 μM), while compounds **3a**, **3b**, and **3g** exhibited moderate activity. Compounds **3d**–**3f**, **3h**, and **3i** were inactive (Table 3). Thus, the introduction of a methyl substituent into the benzene ring of the coumarin fragment (R^4^) led to both an increase in cytotoxicity and an expansion of its spectrum (Figure 14).

**Figure 15:**
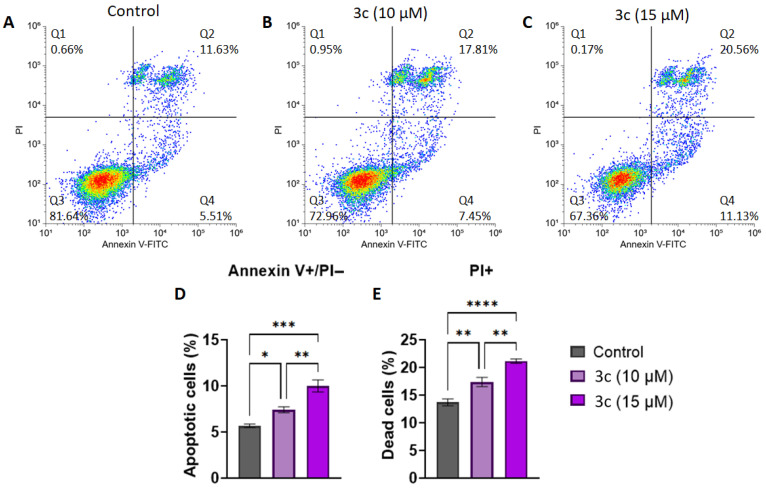
Dot plots of annexin V-FITC and PI-stained Jurkat cells for evaluation of apoptosis induced by 10 μM and 15 μM of **3c** (**A**–**C**); percentages of apoptotic and dead Jurkat cells (**D** and **E**, respectively). Data are presented as mean ± SEM, *n* = 4. Adjusted *p*-values: * *p* ≤ 0.05, ** *p* ≤ 0.01, *** *p* ≤ 0.001, and **** *p* ≤ 0.0001, based on a one-way analysis of variance (ANOVA) with Tukey’s post hoc test.

To examine whether the tested compounds induce cell death via apoptosis, annexin V and propidium iodide were used (Figure 15). Jurkat cells were exposed to **3c**, the most active among the tested compounds, for 72 h, and then flow cytometry analysis was conducted. Annexin V binds phosphatidylserine residues, which are asymmetrically distributed toward the inner plasma membrane, and migrate to the outer plasma membrane during apoptosis. The data show that **3c**, at concentrations of 10 μM and 15 μM, induces dose-dependent apoptotic cell death of Jurkat cells. The dose-dependent increase in dead cells was also shown (Figure 15E and Figures S28–S30 in Supplementary). Doxorubicin was chosen as a positive control (Figure S31 in Supplementary).

**Figure 16:**
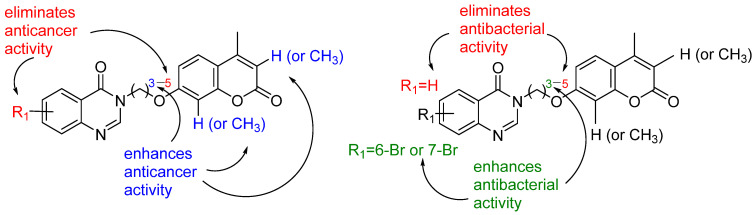
Summary SAR diagram.

The promising antibacterial properties of some derivatives of quinazolinones allow us to consider this core as a privileged scaffold for the design and development of new antibacterial agents [13,26]. For preliminary assessment of antimicrobial potential of obtained quinazolinone–coumarin hybrids, a comparative evaluation of antibacterial properties of derivatives **3a**–**3i** and reference agent levofloxacin (Lev) was performed on a panel of pathogenic Gram-positive and Gram-negative bacteria by the broth microdilution method. The screening panel includes ATCC reference stains and antibiotic-resistant isolates of Gram-positive and Gram-negative bacteria, namely *Staphylococcus aureus* 25923 ATCC, *Staphylococcus epidermidis* 533, *Staphylococcus haemoliticus* 602, *Escherichia coli* 25922 ATCC, *Klebsiella pneumoniae* 13883 ATCC, *Acinetobacter baumanii* 5696, and *Pseudomonas aeruginosa* 27853 ATCC. These bacteria are included in the World Health Organization (WHO) Bacterial Priority Pathogens List, as they are distinguished by high antibiotic resistance. The results of evaluation of antibacterial potency (Table 4) showed that the majority of quinazolinone–coumarin conjugates have bordered antibacterial activity against the tested bacteria strains (MIC 8–16 µg/mL). Moreover, the conjugates with a propylene linker (except for derivative **3a**) were reliably more active than analogs **3h** and **3i** with pentyl chain. The introduction of halogens into the quinazoline fragment in compounds **3d**, **3e**, and **3f** led to the potentiation of antibacterial activity against *Acinetobacter baumanii*. The introduction of an aromatic substituent at position 2 of quinazolin-4(3H)-one **3i** did not result in either anticancer or antibacterial activity. Summary SAR diagram is shown in Figure 16. Thus, the test results indicate the potential for further optimization of the structure and position of functional groups in the heterocyclic moieties of quinazolinone–coumarin conjugates for the development of new antibacterial agents.

## 3. Materials and Methods

### 3.1. Chemical Synthesis

All reagents were obtained in the highest available purity grade from Sigma-Aldrich and Acros Organics and were used without further purification unless otherwise stated. Anhydrous DMF and isopropyl alcohol were purchased from Sigma-Aldrich Co (St. Louis, MO, USA). Anhydrous acetone, 1,2-dichloroethane (DCE), and ethyl acetate (EtOAc) were obtained by distillation over phosphorus pentoxide (P_2_O_5_). Thin-layer chromatography (TLC) was performed on Merck Silicagel 60 F254 plates, and compounds were visualized under UV light (Vilber VL-6.LC). Silica gel (kieselguhr 60–200 μm, 60A, Acros Organics, Geel, Belgium) was used for column chromatography. Yields refer to spectroscopically (^1^H and ^13^C NMR) homogeneous materials. Melting points were measured in sealed capillaries using a Mel-Temp 3.0 instrument (Laboratory Devices Inc., Auburn, AL, USA). NMR spectra were recorded on Bruker Avance 400 MHz spectrometers (400 MHz for ^1^H and 100 MHz for ^13^C) in DMSO-d_6_ or CDCl_3_ with tetramethylsilane (TMS) as an internal standard.

High-resolution mass spectra (HRMS) were obtained on a Bruker Daltonics micrOTOF II instrument (Billerica, Massachusetts, USA) using electrospray ionization (ESI) in positive-ion detection mode. The instrument parameters were as follows: capillary voltage, 4500 V; mass range, *m/z* 50–3000; internal calibration with ESI Tuning Mix (Agilent, Santa Clara, CA USA); nebulizer pressure, 0.3 bar; flow rate, 3 µL/min; dry nitrogen consumption, 4.0 l/min; interface temperature, 180 °C; and injection with a syringe.

**General method for the preparation of 3-{ω-[(4-methyl-2-oxo-2*H*-chromen-7-yl)oxy]alkyl}quinazolin-4(3*H*)-ones 3a–3i.**

To a mixture of quinazolin-4(3H)-one **1a**–**1d** or **4** (4.8 mmol) and of potassium carbonate (0.86 g, 6.2 mmol) in DMF (10 mL), 4.8 mmol of 7-(ω-bromoalkoxy)-4-methyl-2*H*-chromene **2a**–**2e** was added, with stirring. The resulting mixture was stirred at 80 °C for 24 h. The reaction mixture was evaporated under reduced pressure; the residue was treated with water (100 mL); and the solid residue was filtered, dried at room temperature, and purified by flash chromatography on silica gel eluting with a 1,2-dichloroethane-methanol (5:1) mixture. Fractions containing the product were combined and evaporated under reduced pressure. The resulting product was recrystallized from a DMF–water (1:1) mixture and repurified using PLC (1 mm Merck Silicagel 60 F254 plates), eluent CH_3_Cl/MeOH (95:5).

^1^H and ^13^C NMR spectra as well as HRMS for target compounds **3a–3i** are shown at Figures S1–S27 (see Supplementary Materials).

**3-{3-[(4-Methyl-2-oxo-2*H*-chromen-7-yl)oxy]propyl}quinazolin-4(3*H*)-one (3a).**

Yield 82%, mp 169–171 °C, R_f_ 0.21 (ethyl acetate/1,2-dichloroethane, 1:1).

^1^H NMR (300 MHz, DMSO-d_6_) δ 2.23 (p, J = 6.4 Hz, 2H, CH_2_), 2.39 (d, J = 1.3 Hz, 3H, CH_3_), 4.18 (td, J = 6.4 and 3.1 Hz, 4H, CH_2_ × 2), 6.20 (d, J = 1.3 Hz, 1H, H^(3)^-chromene), 6.85 (dd, J = 8.8 and 2.5 Hz, 1H, H^(6)^-chromene), 6.91 (d, J = 2.5 Hz, 1H, H^(8)^-chromene), 7.55 (ddd, J = 8.1, 7.1 and 1.2 Hz, 1H, H^(6)^-quinazolin-4-one), 7.66 (m, 2H, H^(5)^-chromene, H^(7)^-quinazolin-4-one), 7.84 (dd, J = 8.5 and 7.1 Hz, 1H, H^(8)^-quinazolin-4-one), 8.16 (dd, J = 8.0 and 1.6 Hz, 1H, H^(5)^-quinazolin-4-one), 8.38 (s, 1H, H^(2)^-quinazolin-4-one).

^13^C NMR (75 MHz, DMSO-d_6_) δ: 18.6, 28.2, 44.3, 66.5, 101.7, 111.6, 112.8, 113.6, 122.1, 126.5, 126.8, 127.4, 127.6, 134.7, 148.4, 148.6, 153.8, 155.1, 160.6, 160.8, 161.8.

HRMS, *m/z*: calculated for 363.1339 [M+H]^+^, 363.1329 found [M+H]^+^

**3-{3-[(3,4-Dimethyl-2-oxo-2*H*-chromen-7-yl)oxy]propyl}quinazolin-4(3*H*)-one (3b).**

Yield 77%, mp 184–186 °C, R_f_ 0.24 (ethyl acetate/1,2-dichloroethane, 1:1).

^1^H NMR (DMSO-d_6_), δ: 2.07 (s, 3H, CH_3_), 2.22–2.27 (m, 2H, CH_2_), 2.35 (s, 3H, CH_3_), 4.17 (m, 4H, CH_2_ × 2), 6.80–6.88 (m, 2H, H^(6)^-chromene, H^(8)^-chromene), 7.54 (t, J = 7.7 Hz, 1H, H^(6)^-quinazolin-4-one), 7.66 (m, 2H, H^(5)^-chromene, H^(7)^-quinazolin-4-one), 7.83 (t, J = 7.7 Hz, 1H, H^(8)^-quinazolin-4-one), 8.15 (d, J = 8.0 Hz, 1H, H^(5)^-quinazolin-4-one) 8.37 (s, 1H, H^(2)^-quinazolin-4-one).

^13^C NMR (DMSO-d_6_), δ: 13.3, 15.3, 28.2, 44.3, 66.4, 101.4, 112.7, 114.2, 118.4, 122.1, 126.5, 126.6, 127.4, 127.6, 134.7, 147.2, 148.5, 148.6, 153.4, 160.8, 161.7.

HRMS, *m/z*: calculated for 377.1496 [M+H]^+^, found 377.1472 [M+H]^+^.

**3-{3-[(4,8-Dimethyl-2-oxo-2*H*-chromen-7-yl)oxy]propyl}quinazolin-4(3*H*)-one (3c).**

Yield 70%, mp 177.5–179 °C, R_f_ 0.27 (ethyl acetate/1,2-dichloroethane, 1:1).

^1^H NMR (DMSO-d_6_), δ: 2.03 (s, 3H, CH_3_), 2.26 (p, J = 6.3 Hz, 2H, CH_2_), 2.38 (d, J = 1.2 Hz, 3H, CH_3_), 4.21 (q, J = 6.1 Hz, 4H, CH_2_ × 2), 6.19 (d, J = 1.2 Hz, 1H, H^(3)^-chromene), 7. 02 (d, 1H, J = 8.0 Hz, H^(6)^-chromene), 7.51–7.59 (m, 2H, H^(5)^-chromene, H^(6)^-quinazolin-4-one), 7.65–7.68 (m, 1H, H^(7)^-quinazolin-4-one), 7.82 (ddd, J = 8.2, 7.1 and 1.6 Hz, 1H, H^(8)^-quinazolin-4-one), 8.15 (m, 1H, H^(5)^-quinazolin-4-one), 8.38 (s, 1H, H^(2)^-quinazolin-4-one).

^13^C NMR (DMSO-d_6_), δ: 8.2, 18.6, 28.6, 44.4, 66.7, 108.5, 111.5, 112.9, 113.8, 122.1, 124.0, 126.5, 127.4, 127.6, 134.7, 148.5, 152.4, 154.0, 159.4, 160.6, 160.8.

HRMS, *m/z*: calculated for 377.1496 [M+H]^+^, 377.1466 found [M+H]^+^.

**6-Chloro-3-{3-[(4-methyl-2-oxo-2*H*-chromen-7-yl)oxy]propyl}quinazolin-4(3*H*)-one (3d).**

Yield 68%, mp 222.5–224.5 °C, R_f_ 0.27 (ethyl acetate/1,2-dichloroethane, 1:1).

^1^H NMR (DMSO-d_6_), δ: 2.22 (q, J = 6.3 Hz, 2H, CH_2_), 2.39 (d, J = 2.2 Hz, 3H, CH_3_), 4.19 (m, 4H, CH_2_ × 2), 6.20 (d, J = 2.1 Hz, 1H, H^(3)^-chromene), 6.82 (dd, J = 8.8 and 2.5 Hz, 1H, H^(6)^-chromene), 6.89 (d, J = 2.5 Hz, 1H, H^(8)^-chromene), 7.64 (d, J = 12 Hz, 1H, H^(5)^-chromene), 7.71 (d, 1H, H^(7)^-quinazolin-4-one), 7.85 (d, J = 2.5 Hz, 1H, H^(8)^-chromene), 8.41 (s, 1H, H^(2)^-quinazolin-4-one).

^13^C NMR (DMSO-d_6_), δ: 18.6, 28.0, 44.5, 66.5, 101.7, 111.6, 112.8, 113.7, 123.3, 125.4, 126.8, 129.9, 131.7, 134.8, 147.2, 149.1, 153.8, 155.1, 159.9, 161.8.

HRMS, *m/z*: calculated for 397.0950 [M+H]^+^, found 397.0939 [M+H]^+^.

**6-Bromo-3-{3-[(4-methyl-2-oxo-2*H*-chromen-7-yl)oxy]propyl}quinazolin-4(3*H*)-one (3e).**

Yield 68%, mp 199–201 °C, R_f_ 0.29 (ethyl acetate/1,2-dichloroethane, 1:1).

^1^H NMR (DMSO-d_6_), δ: 2.22 (q, J = 6.3 Hz, 2H, CH_2_), 2.38 (d, J = 2.2 Hz, 3H, CH_3_), 4.18 (m, 4H, CH_2_ × 2), 6.20 (d, J = 1.4 Hz, 1H, H^(3)^-chromene), 6.82 (dd, J = 8.8 and 2.6 Hz, 1H, H^(6)^-chromene), 6.88 (m, 1H, H^(8)^-chromene), 7.63 (m, 2H, H^(5)^-chromene, H^(7)^-quinazolin-4-one), 7.96 (dd, J = 8.7 and 2.4 Hz, 1H, H^(8)^-quinazolin-4-one), 8.20 (d, 1H, H^(5)^-quinazolin-4-one) 8.43 (s, 1H, H^(2)^-quinazolin-4-one).

^13^C NMR (DMSO-d_6_), δ: 18.6, 28.0, 44.5, 66.5, 101.7, 111.7, 112.8, 113.6, 119.9, 123.7, 126.8, 128.6, 130.0, 137.5, 147.4, 149.2, 153.8, 155.1, 159.7, 160.6, 161.8.

HRMS, *m/z*: calculated for 441.0444 [M+H]^+^, 441.0419 found [M+H]^+^.

**7-Bromo-3-{3-[(4-methyl-2-oxo-2*H*-chromen-7-yl)oxy]propyl}quinazolin-4(3*H*)-one (3f).**

Yield 62%, mp 178–180 °C, R_f_ 0.28 (ethyl acetate/1,2-dichloroethane, 1:1).

^1^H NMR (DMSO-d_6_), δ: 2.22 (q, J = 6.3 Hz, 2H, CH_2_), 2.38 (d, J = 2.0 Hz, 3H, CH_3_), 4.17 (t, J = 6.2 Hz, 4H, CH_2_ × 2), 6.20 (d, J = 1.4 Hz, 1H, H^(3)^-chromene), 6.83 (dd, J = 8.8 and 2.5 Hz, 1H, H^(6)^-chromene), 6.89 (d, 1H, H^(8)^-chromene), 7.64 (d, J = 8.5 Hz, 1H, H^(5)^-chromene), 7.70 (m, 1H, H^(6)^-quinazolin-4-one), 7.88 (d, J = 1.9 Hz, 1H, H^(8)^-quinazolin-4-one), 8.05 (d, J = 8.0 Hz, 1H, H^(5)^-quinazolin-4-one), 8.42 (s, 1H, H^(2)^-quinazolin-4-one).

^13^C NMR (DMSO-d_6_), δ: 18.6, 28.0, 44.5, 66.5, 101.7, 111.6, 112.8, 113.7, 121.2, 126.9, 128.3, 128.6, 129.1, 129.8, 130.5, 149.6, 150.0, 153.8, 155.1, 160.6, 161.8.

HRMS, *m/z*: calculated for 441.0444 [M+H]^+^, 441.0436 found [M+H]^+^.

**3-{3-[(4-Methyl-2-oxo-2*H*-chromen-7-yl)oxy]butyl}quinazolin-4(3*H*)-one (3g).**

Yield 86%, mp 178–179 °C, R_f_ 0.22 (ethyl acetate/1,2-dichloroethane, 1:1).

^1^H NMR (DMSO-d_6_), δ: 1.76–1.94 (m, 4H, CH_2_ × 2), 2.38 (d, J = 2.2 Hz, 3H, CH_3_), 4.06 (t, J = 6.5 Hz, 2H, CH_2_), 4.12 (t, J = 6.5 Hz, 2H, CH_2_), 6.19 (s, J = 1.4 Hz, 1H, H^(3)^-chromene), 6.91–6.94 (m, 2H, H^(6)^-chromene, H^(8)^-chromene), 7.54 (t, J = 12 Hz, 1H, H^(6)^-quinazolin-4-one), 7.65 (m, 2H, H^(5)^-chromene, H^(8)^-quinazolin-4-one), 7.82 (t, J = 8.7 Hz, 1H, H^(7)^-quinazolin-4-one), 8.16 (d, J = 8.0 Hz, 1H, H^(5)^-quinazolin-4-one), 8.42 (s, 1H, H^(2)^-quinazolin-4-one).

^13^C NMR (DMSO-d_6_), δ: 18.6, 25.8, 26.1, 46.1, 68.3, 101.7, 111.6, 112.9, 113.5, 122.0, 126.5, 126.9, 127.4, 127.6, 134.7, 148.4, 148.5, 153.8, 155.2, 160.6, 160.7, 162.1.

HRMS, *m/z*: calculated for 377.1496 [M+H]^+^, 377.1476 found [M+H]^+^.

**3-{5-[(4-Methyl-2-oxo-2*H*-chromen-7-yl)oxy]pentyl}quinazolin-4(3*H*)-one (3h).**

Yield 81%, mp 174–175.5 °C, R_f_ 0.71 (ethyl acetate/1,2-dichloroethane, 1:1).

^1^H NMR (DMSO-d_6_), δ: 1.46 (m, J = 7.3 Hz, 2H, CH_2_), 1.79 (m, J = 7.6 Hz, 4H, CH_2_ × 2), 2.39 (s, J = 1.2 Hz, 3H, CH_3_), 4.05 (dt, J = 8.8 and 6.4 Hz, 4H, CH_2_ × 2), 6.19 (s, J = 1.8 Hz, 1H, H^(3)^-chromene), 6.91–6.94 (m, 2H, H^(6)^-chromene, H^(8)^-chromene), 7.54 (t, J = 8.2 and 1.2 Hz, 1H, H^(6)^-quinazolin-4-one), 7.63 (m, 2H, H^(5)^-chromene, H^(7)^-quinazolin-4-one), 7.82 (dd, J = 8.2 and 1.6 Hz, 1H, H^(8)^-quinazolin-4-one), 8.16 (dd, 1H, J = 7.9 and 1.2 Hz, H^(5)^-quinazolin-4-one) 8.41 (s, 1H, H^(2)^-quinazolin-4-one).

^13^C NMR (DMSO-d_6_), δ: 18.6, 23.0, 28.5, 28.8, 46.2, 68.5, 101.6, 111.5, 112.9, 113.5, 122.0, 126.5, 126.9, 127.4, 127.6, 134.7, 148.4, 148.5, 153.8, 155.2, 160.6, 162.2.

HRMS, *m/z*: calculated for 391.1652 [M+H]^+^, 391.1659 found [M+H]^+^.

**3-{5-[(4-Methyl-2-oxo-2*H*-chromen-7-yl)oxy]pentyl}-2-phenylquinazolin-4(3*H*)-one (3i)**.

Yield 79%, mp 141.5–143.5 °C, R_f_ 0.71 (ethyl acetate/1,2-dichloroethane, 1:1).

^1^H NMR (DMSO-d_6_), δ: 1.70 (m, 2H, CH_2_), 1.89 (m, 2H, CH_2_), 2.00 (m, 2H, CH_2_), 2.04 (s, J = 3.3 Hz, 3H, CH_3_), 4.14 (t, J = 8.8 Hz, 2H, CH_2_), 4.75 (t, J = 6.4 Hz, 2H, CH_2_), 6.18 (m, J = 1.8 Hz, 1H, H^(3)^-chromene), 6.89–6.94 (m, 2H, H^(6)^-chromene, H^(8)^-chromene), 7.52–7.56 and 7.59–7.66 (m, 5H, phenyl), 7.91–7.98 (m, 2H, H^(5)^-chromene, H^(6)^-quinazolin-4-one), 8.15 (d, J = 7.9 Hz, 1H,H^(8)^-quinazolin-4-one), 8.50–8.54 (m, 2H, H^(5)^-quinazolin-4-one, H^(7)^-quinazolin-4-one).

^13^C NMR (DMSO-d_6_), δ: 18.5, 22.6, 28.3, 28.6, 67.1, 68.7, 101.8, 111.6, 112.8, 113.6, 115.2, 123.7, 126.8, 127.5, 128.1, 128.5, 128.9, 131.1, 134.6, 138.0, 151.8, 153.7, 155.3, 159.5, 160.5, 162.3, 166.9.

HRMS, *m/z*: calculated for 467.1965 [M+H]^+^, 467.1929 found [M+H]^+^.

### 3.2. Cytotoxicity Assay

Human leukemia cell lines K562 (CCL-243) and HL-60 (CCL-240) and Jurkat (E6.1) were cultured in RPMI 1640 medium supplemented with 10% fetal bovine serum (FBS) and 2 mM L-glutamine. Human neuroblastoma cells SH-SY5Y (CRL-2266), HeLa (CCL-2) cervical tumor cells, HepG2(HB-8065) hepatocellular carcinoma cell line, and U87(MG) and U251(MG) glioblastoma cell lines were cultured in DMEM medium supplemented with 10% FBS. All cultures were incubated at 37 °C under 5% CO_2_. Cells were seeded in 96-well plates at a density of 1 × 10^4^ cells per well. Compound **3a** was added at concentrations ranging from 0.8 to 100 µM. DMSO (1%), which corresponded to the maximum concentration of the drug, and was used as a control solution. The total volume per well was 150 µL. After 48 h of incubation, 10 μL of resazurin solution (0.5 mg/mL in PBS) was added, and the cells were incubated for an additional 4 h under the same conditions. Fluorescence was measured at 590 nm (excitation at 560 nm) using a CLARIOstar microplate reader (BMG Labtech, Ortenberg, Germany). Cell viability was normalized to that of the control group, and IC_50_ values were calculated by nonlinear regression in GraphPad Prism v9.5.1. Each concentration was tested in at least three replicates.

### 3.3. Flow Cytometry

Jurkat cells were analyzed on a BD LSR Fortessa ™ flow cytometer (Becton Dickinson, Franklin Lakes, New Jersey, USA). Seventy-two hours prior to analysis, cells were seeded on 24-well plates (TPP, Trasadingen, Switzerland) at 5 × 10^5^ cells/well density and then treated with 10 and 15 µM of **3c** compound. Apoptotic cells were detected using annexin V labeled with fluorescein (annexin V-FITC, λ_Ex_/λ_Em_ = 494/518 nm, Thermo Fisher Scientific, Waltham, MA, USA). After incubation with **3c**, according to manufacturer’s instructions, cells were centrifuged (300 rcf, 5 min), the supernatant was removed, and cells were resuspended in annexin binding buffer (10 mM HEPES, 140 mM NaCl, 2.5 mM CaCl_2_, pH 7.4). Then, 5 µL of annexin V-FITC solution was added to 100 µL of the cell suspension (10^6^ cells/mL). Samples were incubated for 15 min in the dark at room temperature. Then, 200 μL of annexin binding buffer was added, and cells were put on ice. To determine cells with an injured membrane, propidium iodide was added to a concentration of 10 μg/mL 40 sec before measurement (PI, λ_Ex_/λ_Em_ = 535/617 nm, Sigma, St. Louis, MO, USA). Cells stained with annexin V, but not PI (lower right quadrant), were considered apoptotic. In turn, cells stained with PI (both upper quadrants) were considered dead because they lacked an intact membrane.

### 3.4. Toxicity Studies in Mice

The animal study was performed in accordance with the European Convention for the Protection of Vertebrate Animals, Directive 86/609/EEC [27], the European Convention for Humane Methods for Animal Welfare and Maintenance [28], and the National Standard of the Russian Federation 33044-2014 “Good Laboratory Practice” [29]. The ethical aspects of animal experimentation were reviewed and approved by the local ethics committee of the Federal State Unitary Enterprise “Research Institute of Hygiene, Toxicology, and Occupational Pathology”, with protocol number 1/2024, dated 13 March 2024. The toxicity of compound **3a** was studied according to [30]. Outbred white mice of both sexes, weighing 25–30 g and aged 12–13 weeks, were used as a biomodel. The experimental and control groups contained 10 animals each, with an equal number of males and females. The total number of animals was 94. Compound **3a** was administered intragastrically through a metal curved feeding needle.

To assess acute toxicity, 99% DMSO was used as the solvent; the maximum achievable dose was 250 mg/kg due to solubility limitations (50 mg/mL). Control animals received only the solvent. The animals were observed continuously for 4 h after administration and periodically for 14 days for signs of toxicity and mortality.

For subchronic and chronic toxicity studies, a 2.5% aqueous starch solution was used as the carrier. Control animals received only 2.5% aqueous starch solution. Body weight was measured using an electronic scale (NP-1000S, A&D, Tokio, Japan). Behavioral analysis was performed using a Laboras automated system (Hoofddorp, The Netherlands), and grip strength was measured using a Bioset GS3 (Barcelona, Spain). Hematological parameters were analyzed using a Micro CC20+ Vet analyzer (North Attleboro, MA, USA). Serum biochemistry was performed using a Chem Well 2910 analyzer (Awareness Technology, Palm City, FL, USA) and DiaSys and Diakon-DS reagent kits (Pushchino, Russia).

After euthanasia, in all experiments, macroscopic pathological and histological examinations were performed on the internal organs (heart, lungs, liver, spleen, thymus, pancreas, kidneys, stomach, and small and large intestines) taken from 8 animals (4 males; 4 females) from each experimental group [31,32]. The biomaterial was fixed in 10% neutral formalin for 4 days. The samples were then dehydrated in a battery of alcohols of ascending strength and cleared in chloroform in a Cytadel 2000 histoprocessor (Shendon, UK) and embedded in Histomix paraffin medium (Biovitrum, St. Peterburg, Russia). Paraffin sections, 4–5 μm thick, were obtained using an HM340E rotary microtome (Microm, Neuss, Germany) and mounted on glass slides. For the overview study, sections were stained with hematoxylin and eosin using the standard method [29]. Microscopic preparations were examined and photographed using an AxioScope A1 microscope (ZEISS, Oberkochen, Germany) equipped with an AxioCam MRc5 high-resolution digital camera. The resulting photographs were processed using ZENpro 2012 software (ZEISS, Oberkochen, Germany).

Statistical analysis was performed using Statistica v10. Quantitative data were analyzed using Student’s *t*-test for independent samples. Differences were considered significant at *p* < 0.05 [33].

### 3.5. Evaluation of Antibacterial Properties

Testing of antibacterial activity of the new quinazolin-4(3H)-one–coumarin hybrids **3a**–**3i** was performed on a panel of clinical isolates of Gram-positive microorganisms: *S. aureus 25923 ATCC*, *S. epidermidis* 533, *S. haemoliticus* 602 and the Gram-negative strain *E.coli* 25922 ATCC, *Klebsiella pneumoniae* 13883 ATCC, *Acinetobacter baumanii* 5696, and *Pseudomonas aeruginosa* 27853 ATCC, as described earlier [34].

A comparative assessment of the spectrum of antibacterial action on reference strains of Gram-positive and Gram-negative microorganisms was carried out using a microdilutions method for determining the minimum inhibitory concentration (MIC) by serial dilutions (in Mueller–Hinton broth, saline solution, and Hanks’ solution) using 96-well sterile plates [35,36]. Stock solutions of the tested compounds in DMSO were prepared at a concentration of 1 mg/mL. The control antibiotic, Levofloxacin, was used at a concentration of 1 mg/mL. The MIC was defined as the minimum concentration of the test compounds that completely inhibited visible growth of the microbial strains. It should be noted that each experiment included a broth and bacterial culture growth control.

To prepare the inoculum, a pure 24 h culture of Gram-positive and Gram-negative microorganisms grown on a solid nutrient medium appropriate for each microorganism type was used. A suspension of the microorganisms was prepared in sterile isotonic sodium chloride solution, bringing the inoculum density to 0.5 according to the McFarland standard (1.5 × 10^8^ CFU/mL). The resulting inoculum was then diluted to a concentration of 5 × 10^5^ CFU/mL with Mueller–Hinton broth. The inoculum was used within 15 min of preparation; the purity of the bacterial strains was checked before each experiment.

To the wells of each plate, 100 μL of Mueller–Hinton broth, saline, or Hanks’ solution was added; the test substance was added to the first well at a concentration of 128 μg/mL, and levofloxacin at 64 μg/mL. The concentration was adjusted to 0.13 μg/mL (for **3a**–**3i**) and 0.06 μg/mL (for levofloxacin) in a volume of 100 μL and serial two-fold dilutions. The prepared inoculum was then added to each well, thereby doubling the concentration of the test compounds. Each drug was titrated twice in the experiment. Wells containing no test substances served as controls (culture growth control). In addition, the purity of the nutrient media and solvents was monitored. The plates were incubated in a thermostat at 36 °C for 24 h. Culture growth was assessed visually by comparing microbial growth in the presence of the test compounds with that in the absence of the test compounds.

## 4. Conclusions

The parent compound **3a** exhibited moderate cytotoxic activity against neuroblastoma and leukemia cells. Evaluation of the cytotoxicity, acute, subchronic, and chronic toxicity of the base compound **3a** revealed the need for structural modification to improve its pharmacological properties. Among the synthesized derivatives, a new lead-compound **3c** with more pronounced cytotoxicity and compounds **3d**–**3g** exhibiting antibacterial activity against *Acinetobacter baumanii* were obtained. Thus, we demonstrated the potential of conjugates containing quinazolin-4(3H)-one and coumarin fragments, as well as the possibility of influencing the direction of biological activity by varying the substituents and the length of the linker.

## Figures and Tables

**Table 1:** Reliable changes in homeostasis parameters in mice with subchronic oral administration of substance **3a**.

Indicators	Exposure Period	Animal Groups	
		3a, 100 mg/kg	Control
**Blood oxygen saturation, %**	14 days	85.2 ± 2.1 *	75.4 ± 4.1
	recovery	99.0 ± 0.0	99.0 ± 0.0
**Number of climbing acts**	14 days	3.3 ± 0.6 *	1.6 ± 0.45
	recovery	1.1 ± 0.4	0.7 ± 0.4
**Average duration of circular rotations, sec.**	14 days	3.8 ± 0.2 *	3.3 ± 0.1
	recovery	3.6 ± 0.2	3.5 ± 0.2
**Average hemoglobin concentration in erythrocytes, g/L**	14 days	483.6 ± 4.1 *	470.3 ± 3.8
	recovery	469.0 ± 4.1	475.1 ± 3.3
**Creatinine, µmol/L**	14 days	31.2 ± 2.8 **	60.2 ± 5.8
	recovery	46.4 ± 7.6	48.1 ± 29.0

Notes * Changes are statistically significant at *p* ≤ 0.05 compared to the average group values of control animals. ** Changes are statistically significant at *p* ≤ 0.05 and are outside the range of values (M ± 2σ) of the corresponding parameters of control animals.

**Table 2:** Reliable changes in homeostasis parameters in mice at different times of chronic intragastric administration of substance **3a**.

Indicators	Duration of Exposure	Animal Groups		
		3a, 4.0 mg/kg	3a, 20.0 mg/kg	Control
**Grip strength, g**	1 month	231.7 ± 7.4 *	224.7 ± 10.8 **	269.1 ± 7.2
	2 months	242.0 ± 10.7	268.1 ± 14.6	270.6 ± 15.3
	Recovery	279.1 ± 9.8	275.7 ± 13.2	283.4 ± 20.4
**Maximum speed of movement, mm/sec**	1 month	161.0 ± 6.4 *	154.9 ± 10.5 *	190.8 ± 9.3
	2 months	169.7 ± 13.5	177.3 ± 6.4	168.1 ± 12.0
	Recovery	158.6 ± 13.4	160.8 ± 5.7	186.4 ± 11.4
**Average speed of movement, mm/sec**	1 month	10.1 ± 1.2	8.6 ± 0.9	11.5 ± 2.0
	2 months	10.3 ± 1.0 **	12.9 ± 1.6	13.3 ± 0.3
	Recovery	9.7 ± 1.76	14.9 ± 2.3	12.8 ± 2.2
**Distance traveled, m**	1 month	3.0 ± 0.4	2.6 ± 0.37	3.5 ± 0.6
	2 months	3.1 ± 0.3 **	3.9 ± 0.5	4.0 ± 0.1
	Recovery	2.9 ± 0.5	4.5 ± 0.7	3.9 ± 0.67
**Number of counterclockwise rotations**	1 month	7.0 ± 0.85	6.3 ± 1.1	6.1 ± 1,4
	2 months	6.4 ± 0.6 *	8.6 ± 1.5	8.8 ± 0,6
	Recovery	7.0 ± 1.3	9.3 ± 1.0	7.6 ± 1.5
**Average speed of circular rotations, mm/sec**	1 month	49.2 ± 2.1	51.1 ± 1.1	49.6 ± 4.2
	2 months	52.0 ± 2.9	48.5 ± 1.5 *	56.6 ± 2.0
	Recovery	52.8 ± 5.2	55.4 ± 2.5	55.6 ± 3.2
**Erythrocytes, ×10^12^**	1 month	12.6 ± 0.5	12.2 ± 0.4 **	13.36 ± 0.19
	2 months	5.9 ± 0.4	7.1 ± 0.7	6.9 ± 0.6
	Recovery	7.3 ± 0.3	7.3 ± 0.6	7.7 ± 0.3
**Platelets, ×10^9^**	1 month	741 ± 51	697 ± 29	781 ± 41
	2 months	623 ± 43	624 ± 38	645 ± 36
	Recovery	611 ± 32 *	626 ± 26 *	759 ± 38
**Thrombocrit, %**	1 month	0.46 ± 0.03	0.46 ± 0.03	0.49 ± 0.02
	2 months	0.39 ± 0.02	0.39 ± 0.02	0.39 ± 0.03
	Recovery	0.38 ± 0.03 *	0.38 ± 0.02 *	0.46 ± 0.03
**Platelet volume distribution width, %**	1 month	11.3 ± 1.1 *	11.7 ± 1.1 *	16.2 ± 1.3
	2 months	9.9 ± 0.3	10.2 ± 0.4	10.1 ± 0.4
	Recovery	10.2 ± 0.3	9.7 ± 0.3	10.4 ± 0.3
**AST activity, IU/L**	1 month	369.9 ± 19.2	340.9 ± 28.6	398.0 ± 7.0
	2 months	211.0 ± 9.0 *	221.0 ± 18.9 **	159.0 ± 11.1
	Recovery	278.0 ± 21.0	285.2 ± 26.1	231.3 ± 14.1
**ALT activity, IU/L**	1 month	116.0 ± 7.1 **	119.1 ± 9.0 **	81.9 ± 6.5
	2 months	80.2 ± 3.0 *	89.0 ± 4.2 *	113.0 ± 8.1
	Recovery	84.2 ± 2.0	84.3 ± 2.0	80.1 ± 6.2
**Urea, mmol/L**	1 month	6.3 ± 0.3	7.3 ± 0.6	6.5 ± 0.3
	2 months	5.2 ± 0.3	4.2 ± 0.2	4.9 ± 0.4
	Recovery	5.5 ± 0.4	4.7 ± 0.3 *	5.7 ± 0.4
**Cholesterol, mmol/L**	1 month	2.2 ± 0.1	2.6 ± 0.2 *	2.1 ± 0.1
	2 months	2.5 ± 0.1 **	2.5 ± 0.2 **	1.6 ± 0.2
	Recovery	2.3 ± 0.1	2.8 ± 0.3	2.3 ± 0.1

* Changes are statistically significant at *p* ≤ 0.05 compared to the average group values of control animals. ** Changes are statistically significant at *p* ≤ 0.05 and are outside the range of values (M ± 2σ) of the corresponding parameters of control animals.

**Table 3:** Antiproliferative activity of compounds **3a–3i** on tumor cell cultures.

Comp.	IC_50_, µM							
	K562	HL60	Jurkat	SHSY5Y	HeLa	HepG2	U87	U251
**3a**	>50	>50	27.0 ± 1.5	27.7 ± 0.8	>50	>50	>50	>50
**3b**	27.2 ± 1.2	>100	30.1 ± 1.3	>100	>50	>50	>50	45 ± 1.2
**3c**	21.1 ± 0.9	47.0 ± 1.1	11.3 ± 1.2	30.1 ± 1.2	17 ± 1.5	>50	16 ± 1.5	15 ± 1.5
**3d**	>50	>50	>50	>50	>50	>50	>50	>50
**3e**	>100	>100	>50	>50	>50	>50	>50	>50
**3f**	>100	>100	>50	>100	>50	>50	>50	>50
**3g**	>50	>50	33.5 ± 1.1	>100	>50	>50	>50	>50
**3h**	>100	>100	>100	>100	>100	>100	>100	>100
**3i**	>100	>100	>100	>100	>100	>100	>100	>100
FUDR	1.3 ± 0.2	1.0 ± 0.3	0.5 ± 0.1	1.5 ± 0.2	>50	>100	>100	>100

**Table 4:** Antibacterial activity (MIC, µg/mL) of compounds **3a**–**3i**.

Bacterial Strains	Compound/MIC, µg/mL									
	3a	3b	3c	3d	3e	3f	3g	3h	3i	Lev
*Staphylococcus aureus* 25923 ATCC	≥64	16	16	16	16	16	16	≥64	≥64	0.25
*Staphylococcus epidermidis* 533	≥64	16	16	16	16	16	16	≥64	≥64	0.13
*Staphylococcus haemoliticus* 602	≥64	16	16	16	16	16	16	≥64	≥64	0.25
*Escherichia coli* 25922 ATCC	≥64	16	16	16	16	16	16	≥64	≥64	0.13
*Klebsiella pneumoniae* 13883 ATCC	≥64	16	16	16	16	16	16	≥64	≥64	0.13
*Acinetobacter baumanii* 5696	64	16	16	16	8	8	16	≥64	≥64	8.0
*Pseudomonas aeruginosa* 27853 ATCC	≥64	16	16	16	16	16	16	≥64	≥64	0.5

## Data Availability

All data generated or analyzed during this study are included in this published article.

## Supplementary Materials

The following supporting information can be downloaded at https://www.mdpi.com/article/10.3390/ijms27104485/s1.
