# Supplementary material for: Synthesis and Biological Evaluation of New Quinazolin-4(3H)-One–Coumarin Hybrids Designed as Anticancer and Antibacterial Agents
Source: Int J Mol Sci. 2026 May 16;27(10):4485. doi: 10.3390/ijms27104485 (PMC13207889; doi:10.3390/ijms27104485)
Supplement: Supplementary file 1 [file ijms-27-04485-s001.zip › ijms-4207384-supplementary.pdf]

Supplementary Material

Synthesis and biological evaluation of new quinazolin-4(3H)-one–coumarin hybrids designed as anticancer and antibacterial agents

Maria P. Paramonova [1], Mikhail S. Novikov [1], Vera A. Sokhraneva [2], Iulia S. Zhivotova [2,3], Vasiliy A. Kezin [2], Martin A. Zenov [2], Irina Yu. Petrushanko [2], Olga N. Novikova [4], Andrey V. Gorshenin [4], Yulia I. Velikorodnaya [1,4], Elena B. Isakova [5], Andrey E. Shchekotikhin [5], Sergey N. Kochetkov [2], Elena S. Matyugina [2], Anastasia L. Khandazhinskaya [2,*]

[1] Volgograd State Medical University, Volgograd, 400131 Russia
[2] Engelhardt Institute of Molecular Biology, Russian Academy of Sciences, Moscow, 119991 Russia
[3] Moscow Center for Advanced Studies, Moscow, 123592 Russia
[4] Federal State Unitary Enterprise "Research Institute of Hygiene, Toxicology, and Occupational Pathology" of the Federal Medical and Biological Agency of the Russian Federation, Volgograd, 400048 Russia
[5] Gause Institute of New Antibiotics, Moscow 119021 Russia.
* Correspondence: khandazhinskaya@bk.ru;

Table of Contents Page | Page

**S1**: ^{1}H-NMR spectrum (400 MHz, DMSO-d6) of compound **3a**	3
**S2**: ^{13}C-NMR spectrum (100 MHz, DMSO-d6) of compound **3a**	3
**S3**: HRMS spectrum of compound **3a**	4
**S4**: ^{1}H-NMR spectrum (400 MHz, DMSO-d6) of compound **3b**	5
**S5**: ^{13}C-NMR spectrum (100 MHz, DMSO-d6) of compound **3b**	5
**S6**: HRMS spectrum of compound **3b**	6
**S7**: ^{1}H-NMR spectrum (400 MHz, DMSO-d6) of compound **3c**	7
**S8**: ^{13}C-NMR spectrum (100 MHz, DMSO-d6)of compound **3c**	7
**S9**: HRMS spectrum of compound **3c**	8
**S10**: ^{1}H-NMR spectrum (400 MHz, DMSO-d6) of compound **3d**	9
**S11**: ^{13}C-NMR spectrum (100 MHz, DMSO-d6) of compound **3d**	9
**S12**: HRMS spectrum of compound **3d**	10
**S13**: ^{1}H-NMR spectrum (400 MHz, DMSO-d6) of compound **3e**	10
**S14**: ^{13}C-NMR spectrum (100 MHz, DMSO-d6) of compound **3e**	11
**S15**: HRMS spectrum of compound **3e**	12
**S16**: ^{1}H-NMR spectrum (400 MHz, DMSO-d6) of compound **3f**	13
**S17**: ^{13}C-NMR spectrum (100 MHz, DMSO-d6) of compound **3f**	13
**S18**: HRMS spectrum of compound **3f**	14
**S19**: ^{1}H-NMR spectrum (400 MHz, DMSO-d6) of compound **3g**	15
**S20**: ^{13}C-NMR spectrum (100 MHz, DMSO-d6) of compound **3g**	15
**S21**: HRMS spectrum of compound **3g**	16

**S22**: ^{1}H-NMR spectrum (400 MHz, DMSO-d6) of compound **3h**	17
**S23**: ^{13}C-NMR spectrum (100 MHz, DMSO-d6) of compound **3h**	17
**S24**: HRMS spectrum of compound **3h**	18
**S25**: ^{1}H-NMR spectrum (400 MHz, DMSO-d6) of compound **3i**	19
**S26**: ^{13}C-NMR spectrum (100 MHz, DMSO-d6) of compound **3i**	19
**S27**: HRMS spectrum of compound **3i**	20
**S28**: Dot plots of Annexin V-FITC and PI stained control Jurkat cells	21
**S29**: Dot plots of Annexin V-FITC and PI stained Jurkat cells for evaluation of apoptosis induced by 10 μM of **3c**	22
**S30**: Dot plots of Annexin V-FITC and PI stained Jurkat cells for evaluation of apoptosis induced by 15 μM of **3c**.	23
**S31**: Dot plots of Annexin V-FITC and PI stained Jurkat cells for evaluation of apoptosis induced by 0.25 μM of doxorubicin.	24

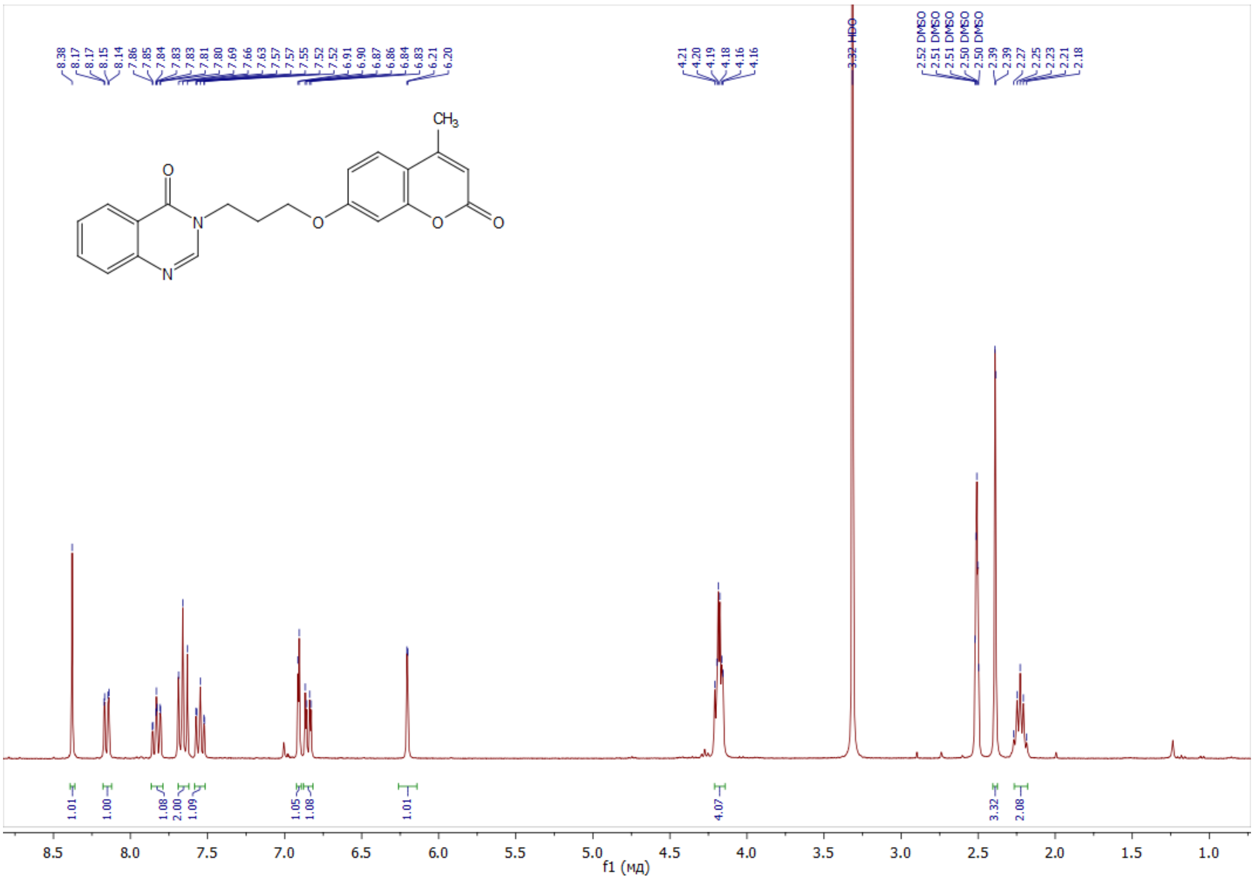

Figure S1 ^{1}H NMR spectrum of compound **3a** in DMSO-*d*6

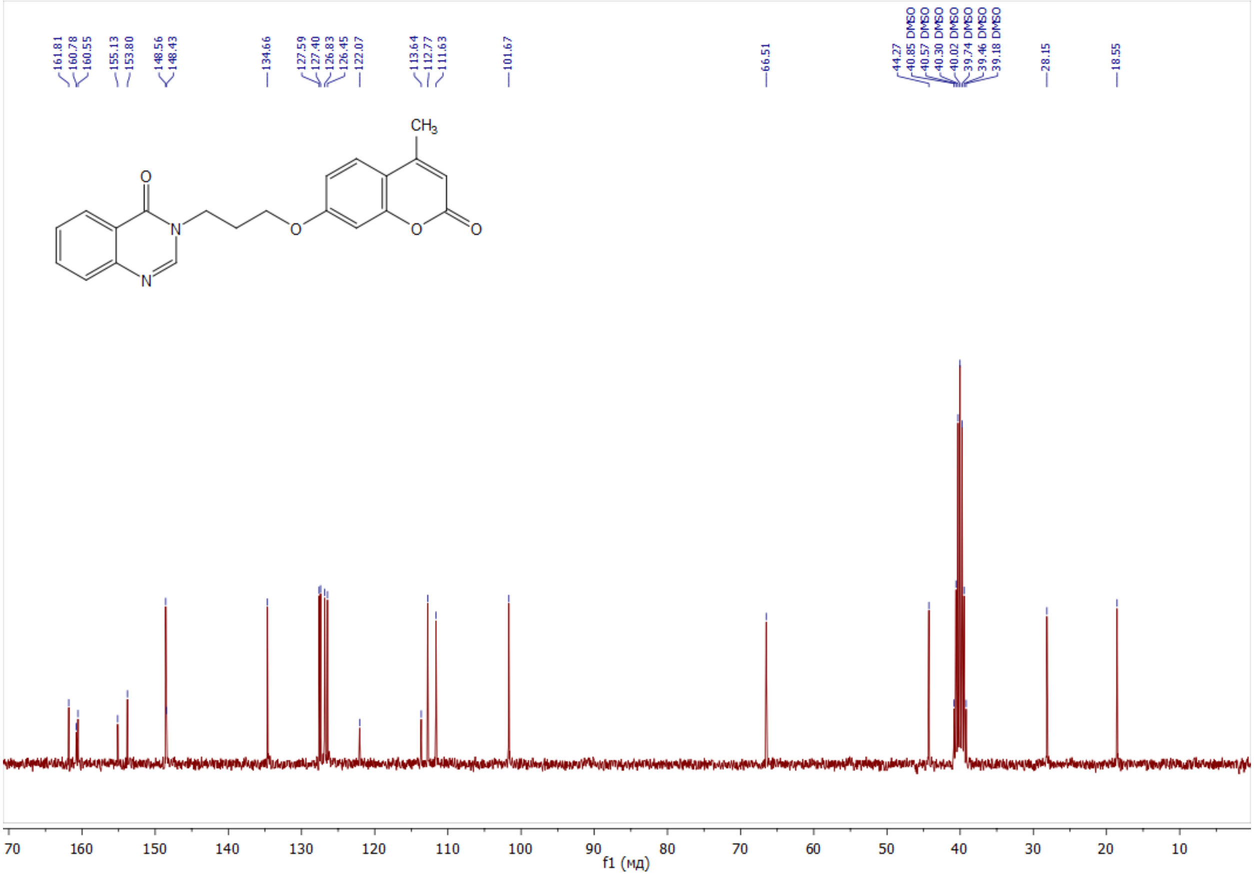

Figure S2 ^{13}C NMR spectrum of compound **3a** in DMSO-*d*6

Display Report

Analysis Info

Analysis Name	D:\Data\Ikar\2026 marth\PDweek\Z 880 300326_22_01_6399.d	Acquisition Date	3/30/2026 1:10:10 PM
Method	ik-lowsmall-2000-positive.m	Operator	BDAL@DE
Sample Name	Z 880 300326	Instrument	compact 8255754.20088
Comment			

Acquisition Parameter

Source Type	ESI	Ion Polarity	Positive	Set Nebulizer	0.4 Bar
Focus	Active	Set Capillary	4500 V	Set Dry Heater	180 °C
Scan Begin	50 m/z	Set End Plate Offset	-500 V	Set Dry Gas	4.0 l/min
Scan End	2000 m/z	Set Charging Voltage	2000 V	Set Divert Valve	Source
		Set Corona	0 nA	Set APCI Heater	0 °C

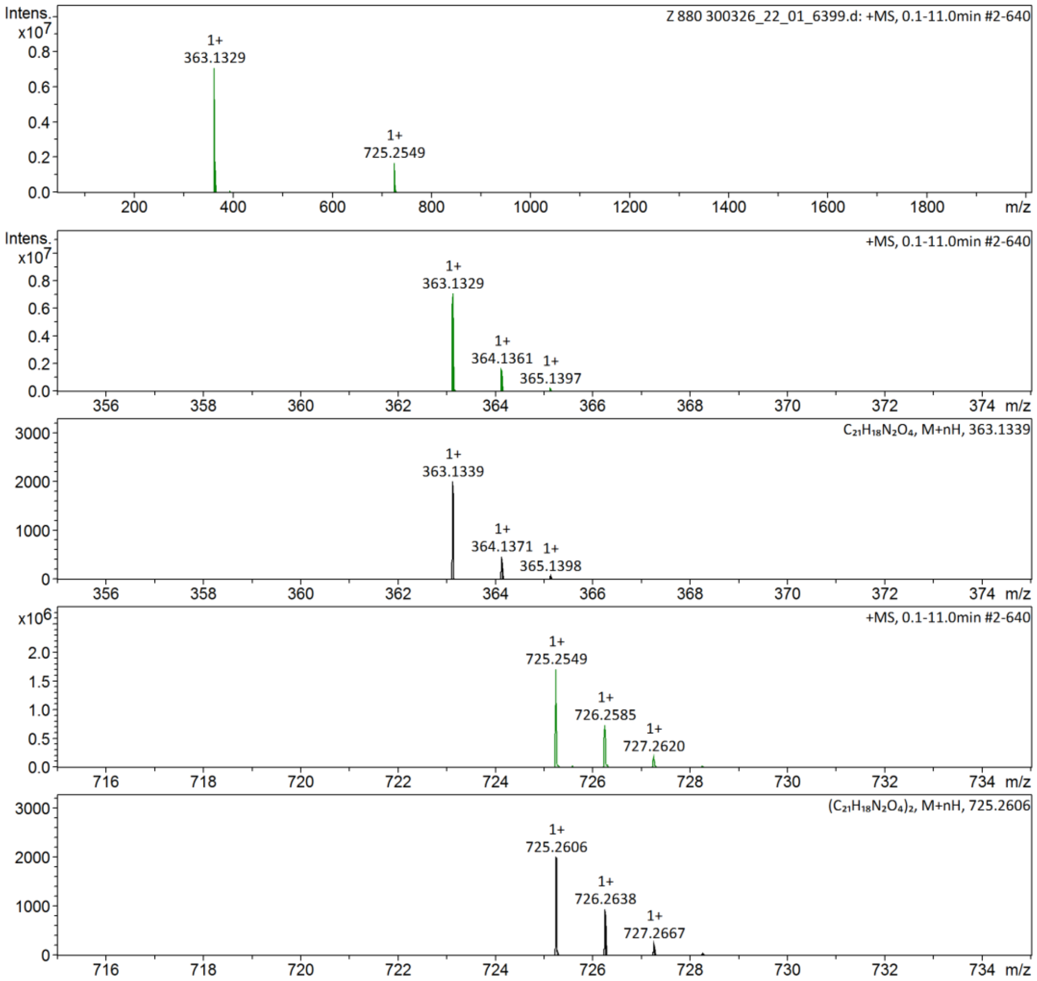

Z 880 300326_22_01_6399.d

Bruker Compass DataAnalysis 4.3 printed: 4/2/2026 11:56:08 AM by: BDAL@DE Page 1 of 1

Figure S3: HRMS spectrum of compound **3a**

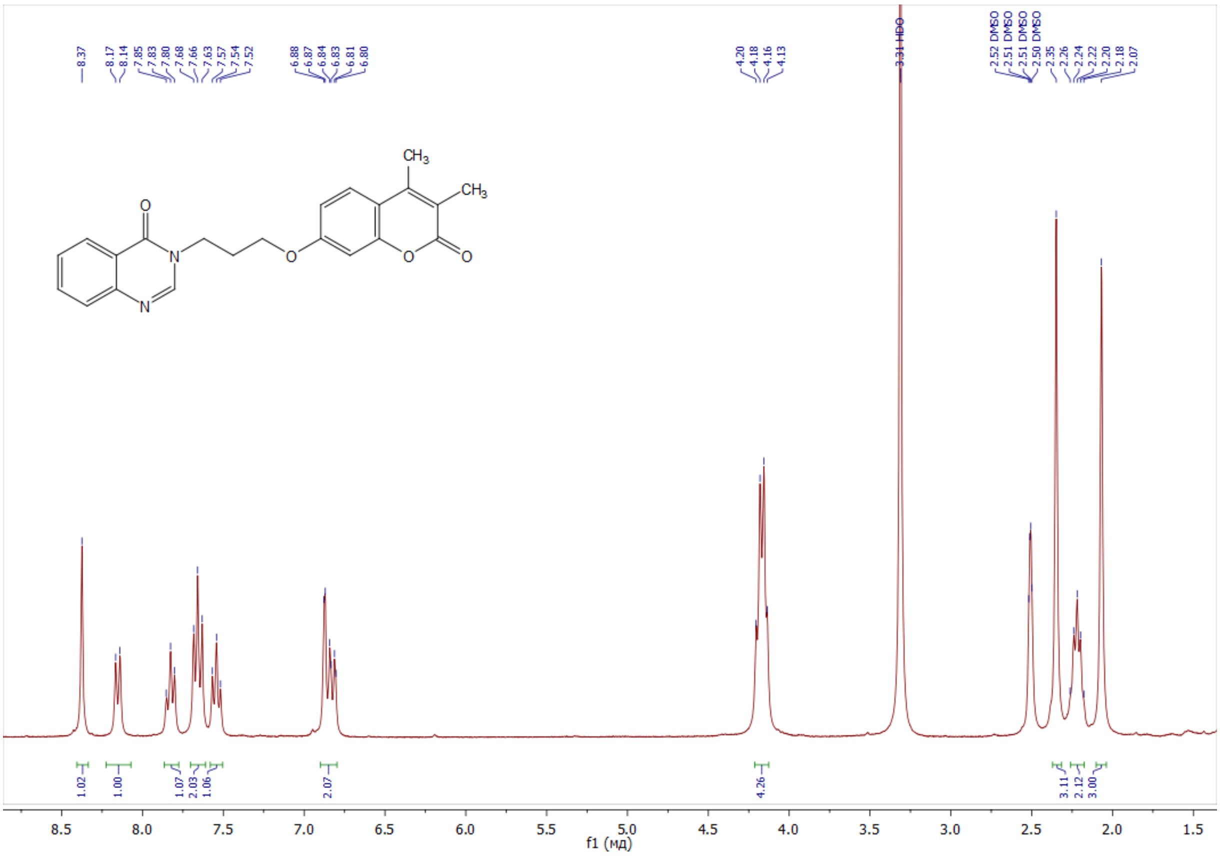

Figure S4 ^{1}H NMR spectrum of compound **3b** in DMSO-*d*6

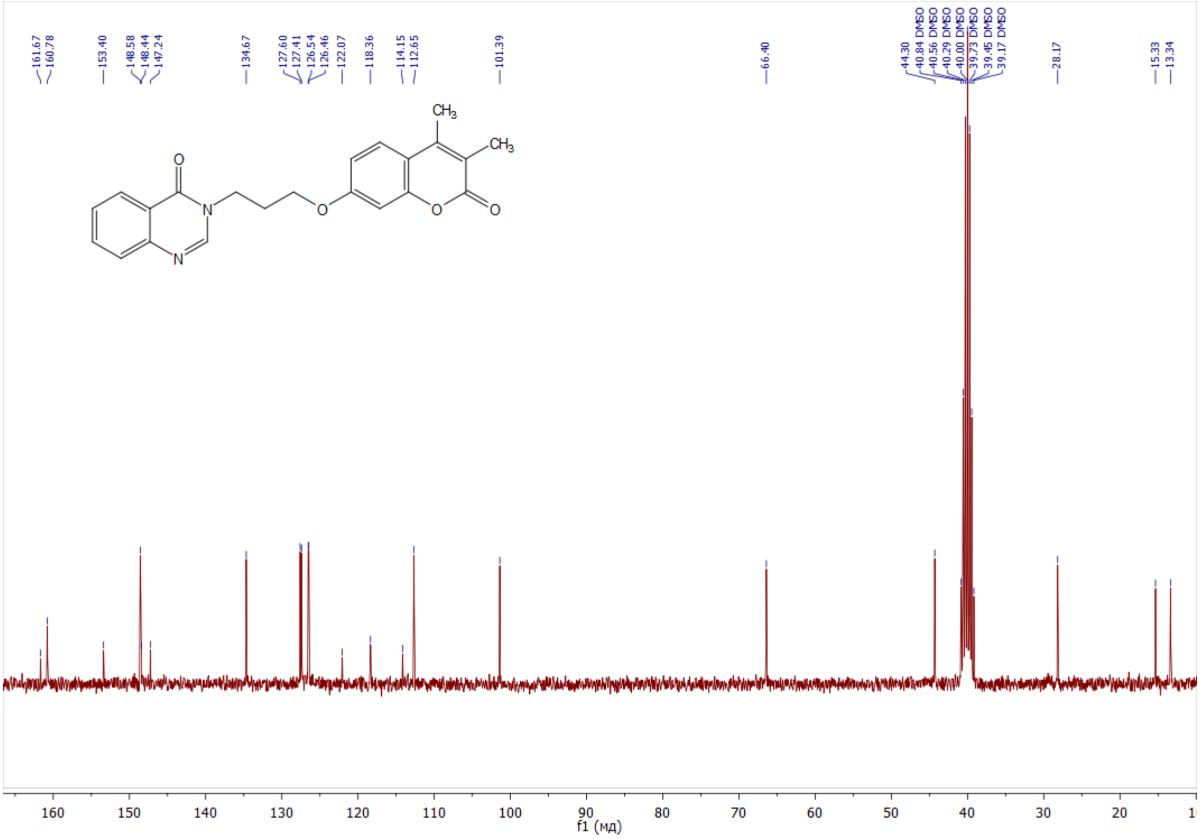

Figure S5 ^{13}C NMR spectrum of compound **3b** in DMSO-*d*6

Display Report

Analysis Info

Analysis Name	D:\Data\Ikar\2026 marth\PDweek\Z 1191 300326_26_01_6403.d	Acquisition Date	3/30/2026 2:24:26 PM
Method	ik-lowsmall-2000-positive.m	Operator	BDAL@DE
Sample Name	Z 1191 300326	Instrument	compact 8255754.20088
Comment			

Acquisition Parameter

Source Type	ESI	Ion Polarity	Positive	Set Nebulizer	0.4 Bar
Focus	Active	Set Capillary	4500 V	Set Dry Heater	180 °C
Scan Begin	50 m/z	Set End Plate Offset	-500 V	Set Dry Gas	4.0 l/min
Scan End	2000 m/z	Set Charging Voltage	2000 V	Set Divert Valve	Source
		Set Corona	0 nA	Set APCI Heater	0 °C

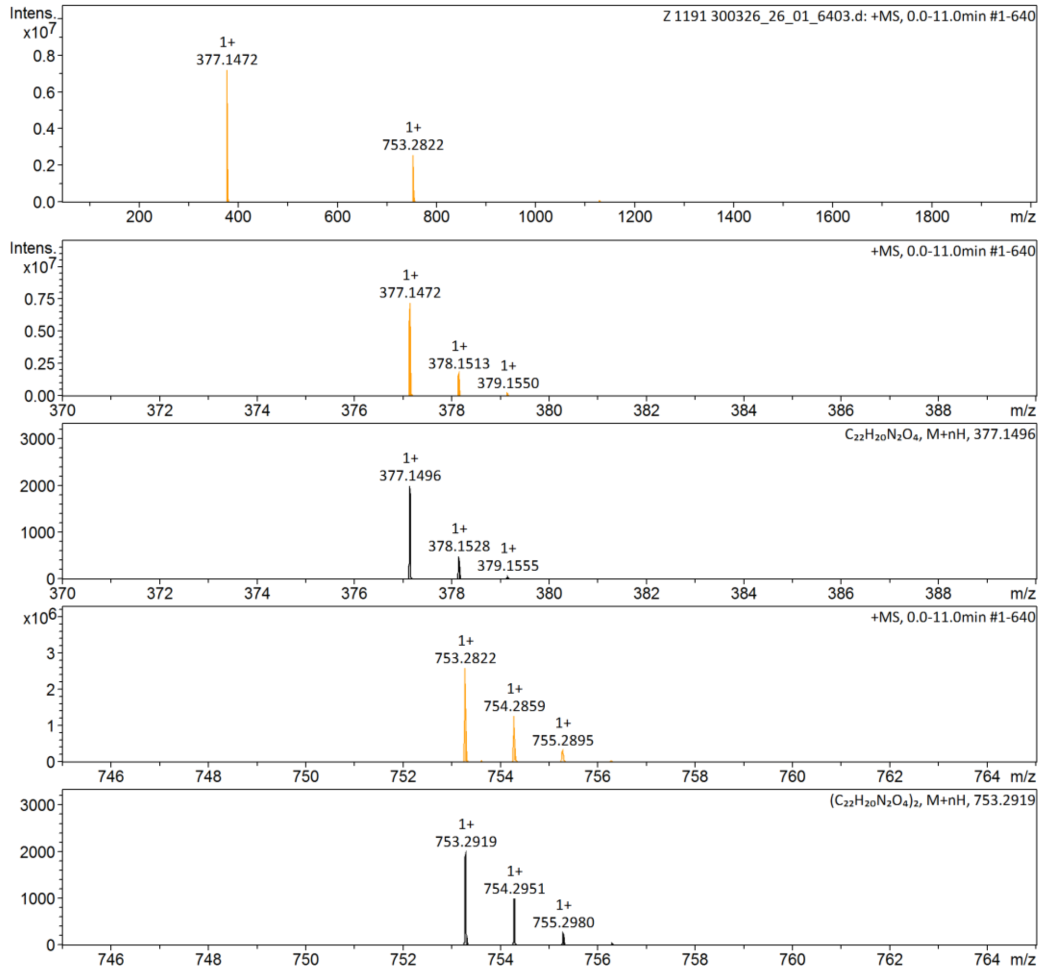

Z 1191 300326_26_01_6403.d

Bruker Compass DataAnalysis 4.3 printed: 4/2/2026 11:42:04 AM by: BDAL@DE Page 1 of 1

Figure S6: HRMS spectrum of compound **3b**

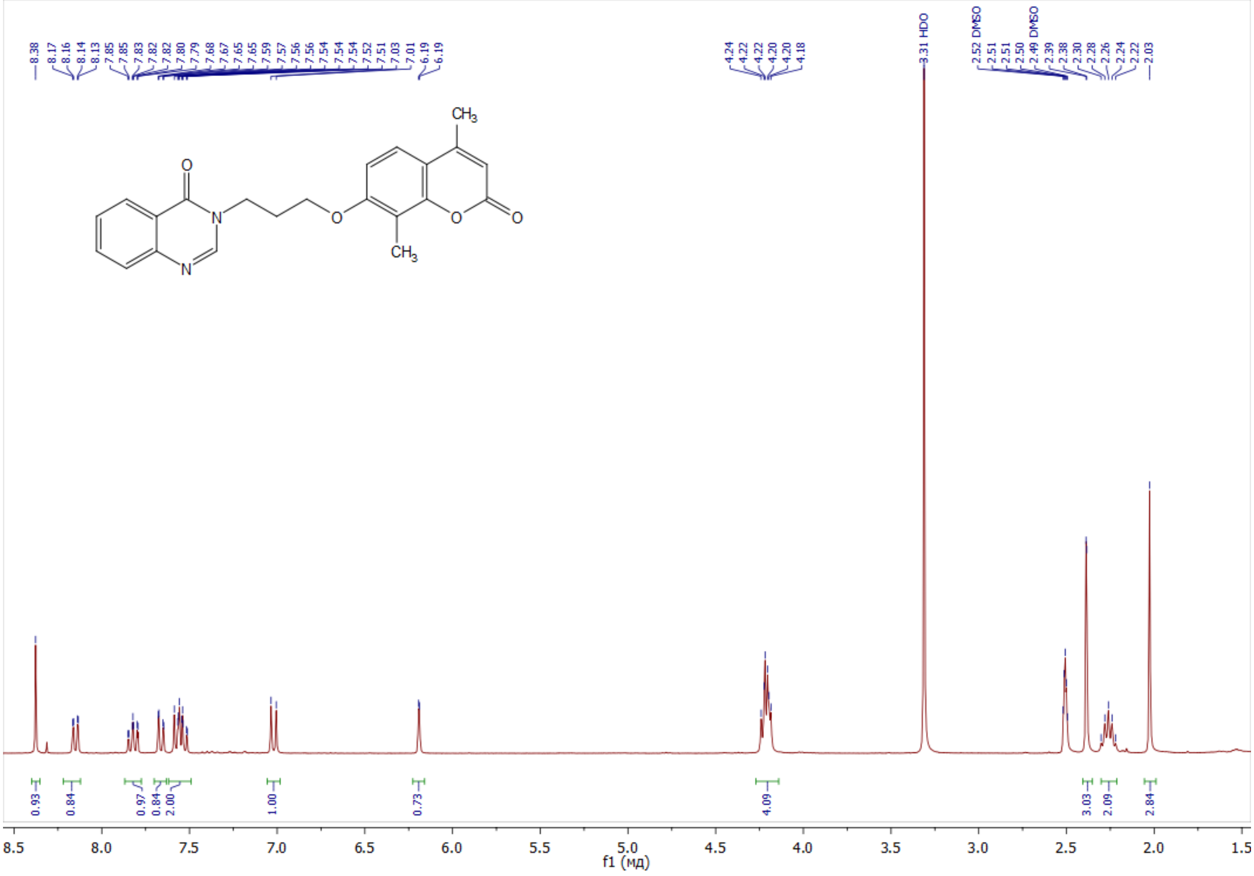

Figure S7 ^{1}H NMR spectrum of compound **3c** in DMSO-*d*6

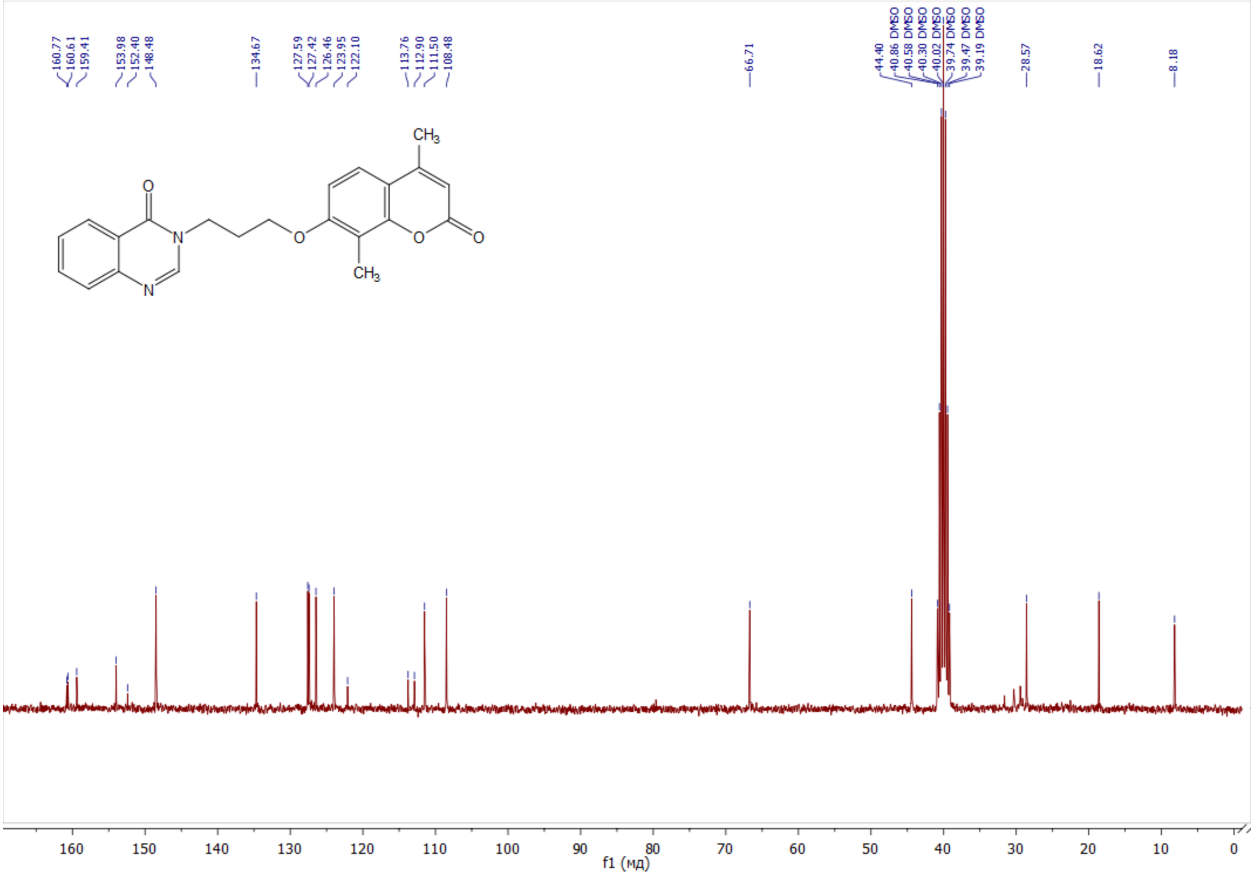

Figure S8 ^{13}C NMR spectrum of compound **3c** in DMSO-*d*6

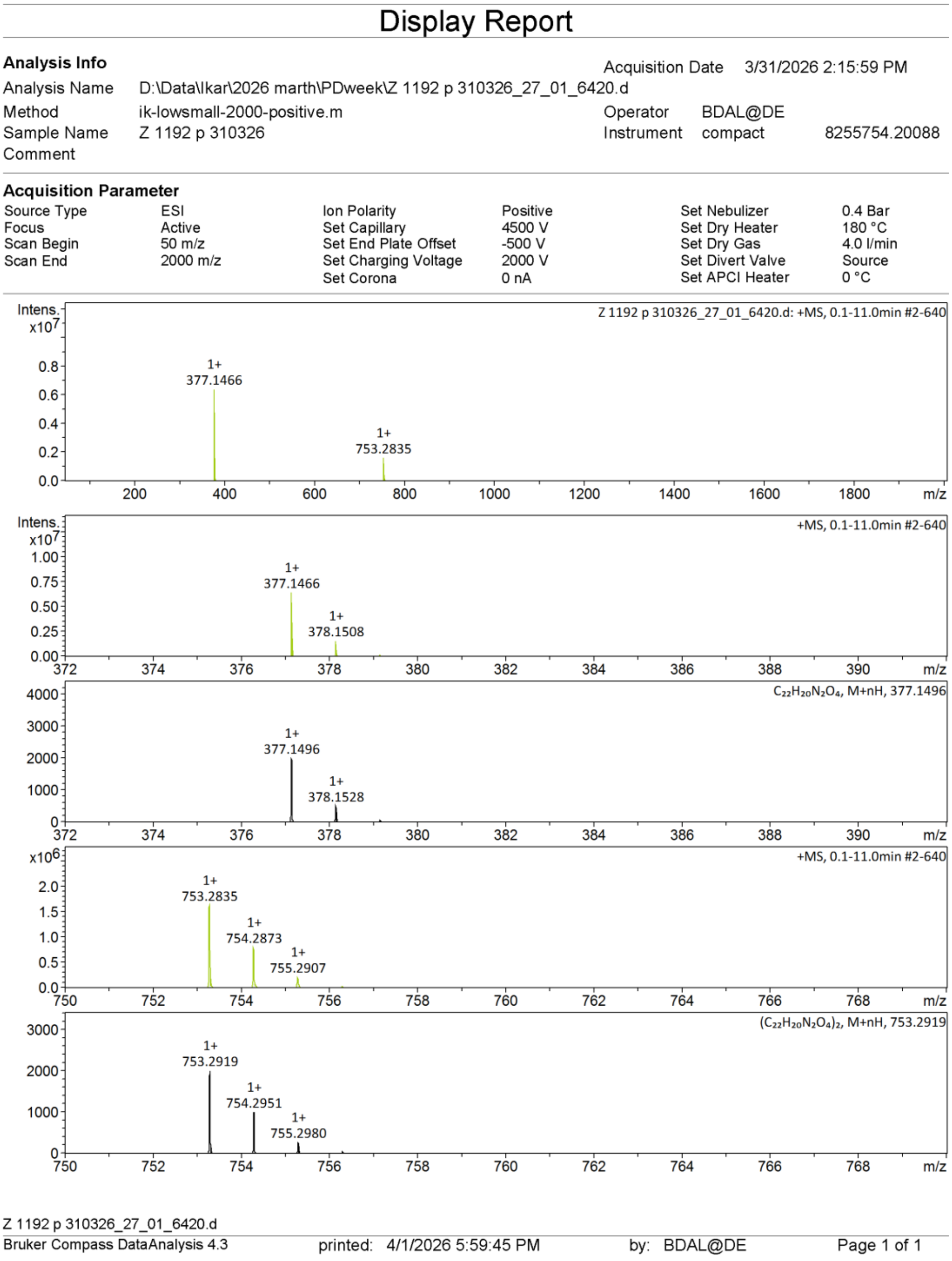

Figure S9: HRMS spectrum of compound **3c**

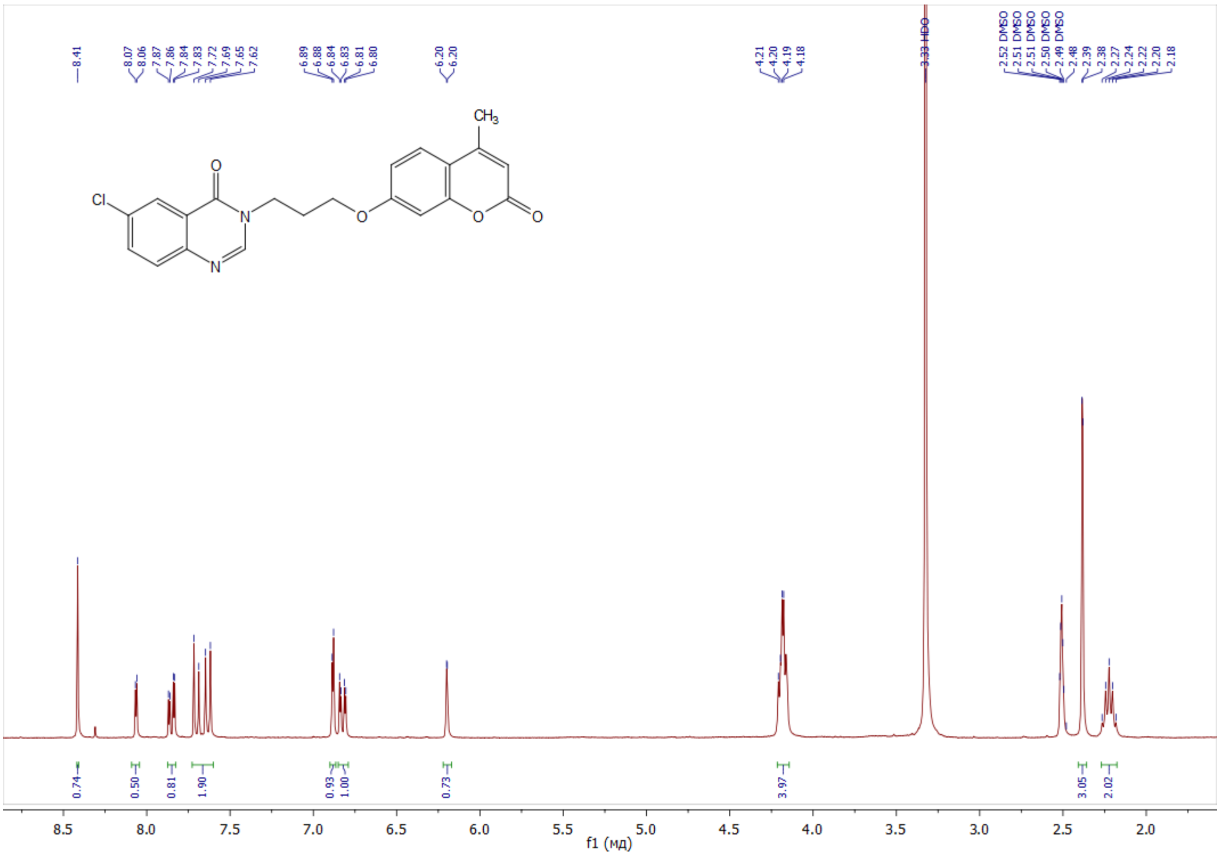

Figure S10 ^{1}H NMR spectrum of compound **3d** in DMSO-*d*6

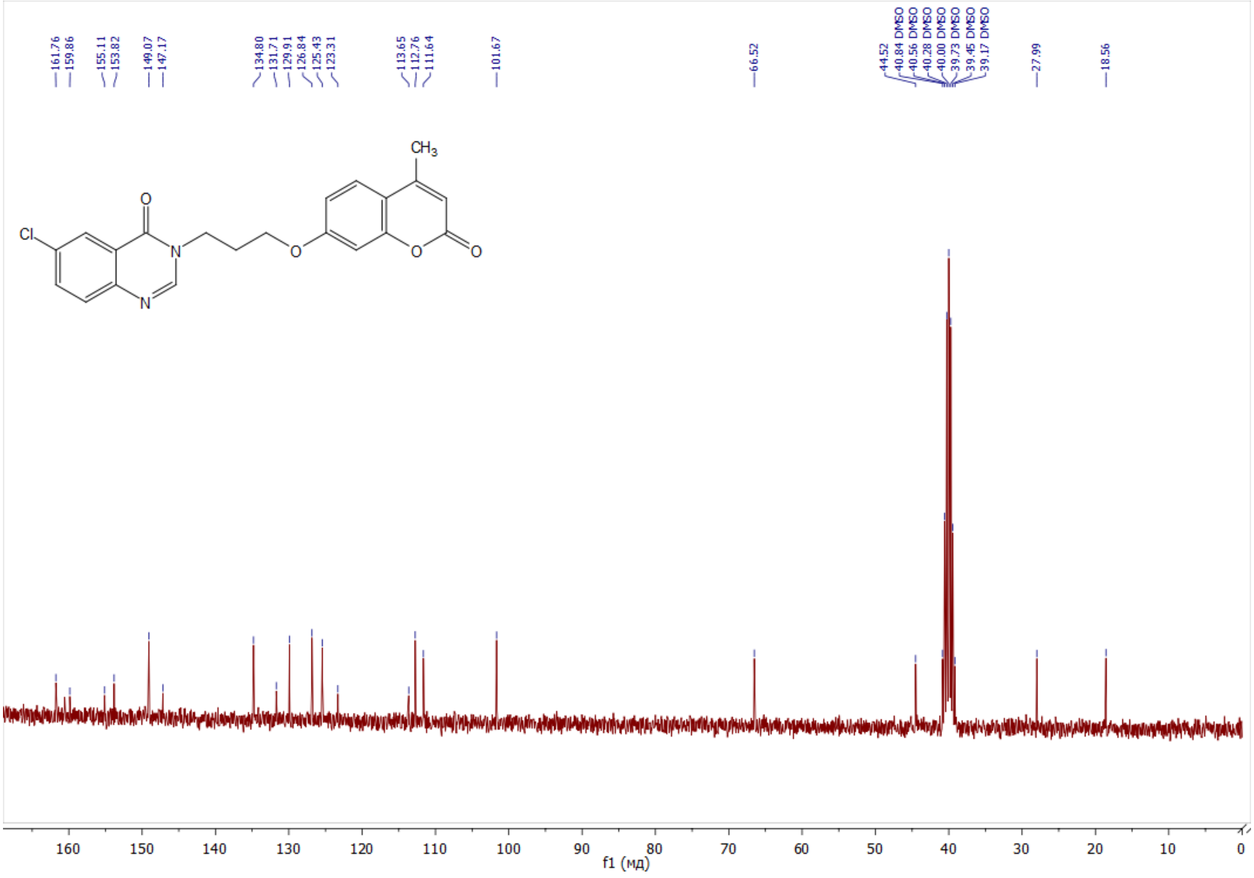

Figure S11 ^{13}C NMR spectrum of compound **3d** in DMSO-*d*6

Display Report

Analysis Info

Analysis Name	D:\Data\Ikar\2026 marth\PDweek\Z 1199 300326_30_01_6407.d	Acquisition Date	3/30/2026 3:38:36 PM
Method	ik-lowsmall-2000-positive.m	Operator	BDAL@DE
Sample Name	Z 1199 300326	Instrument	compact 8255754.20088
Comment			

Acquisition Parameter

Source Type	ESI	Ion Polarity	Positive	Set Nebulizer	0.4 Bar
Focus	Active	Set Capillary	4500 V	Set Dry Heater	180 °C
Scan Begin	50 m/z	Set End Plate Offset	-500 V	Set Dry Gas	4.0 l/min
Scan End	2000 m/z	Set Charging Voltage	2000 V	Set Divert Valve	Source
		Set Corona	0 nA	Set APCI Heater	0 °C

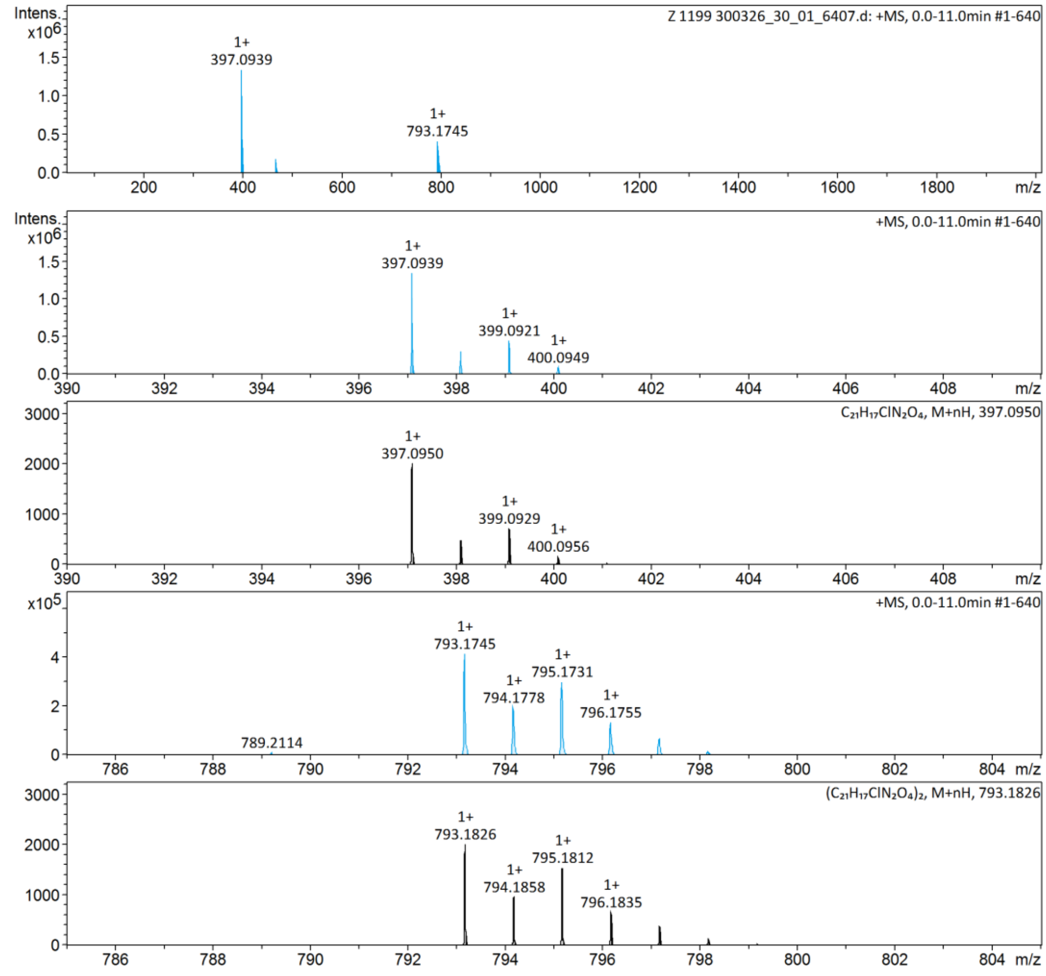

Z 1199 300326_30_01_6407.d

Bruker Compass DataAnalysis 4.3 printed: 4/2/2026 12:03:54 PM by: BDAL@DE Page 1 of 1

Figure S12: HRMS spectrum of compound **3d**

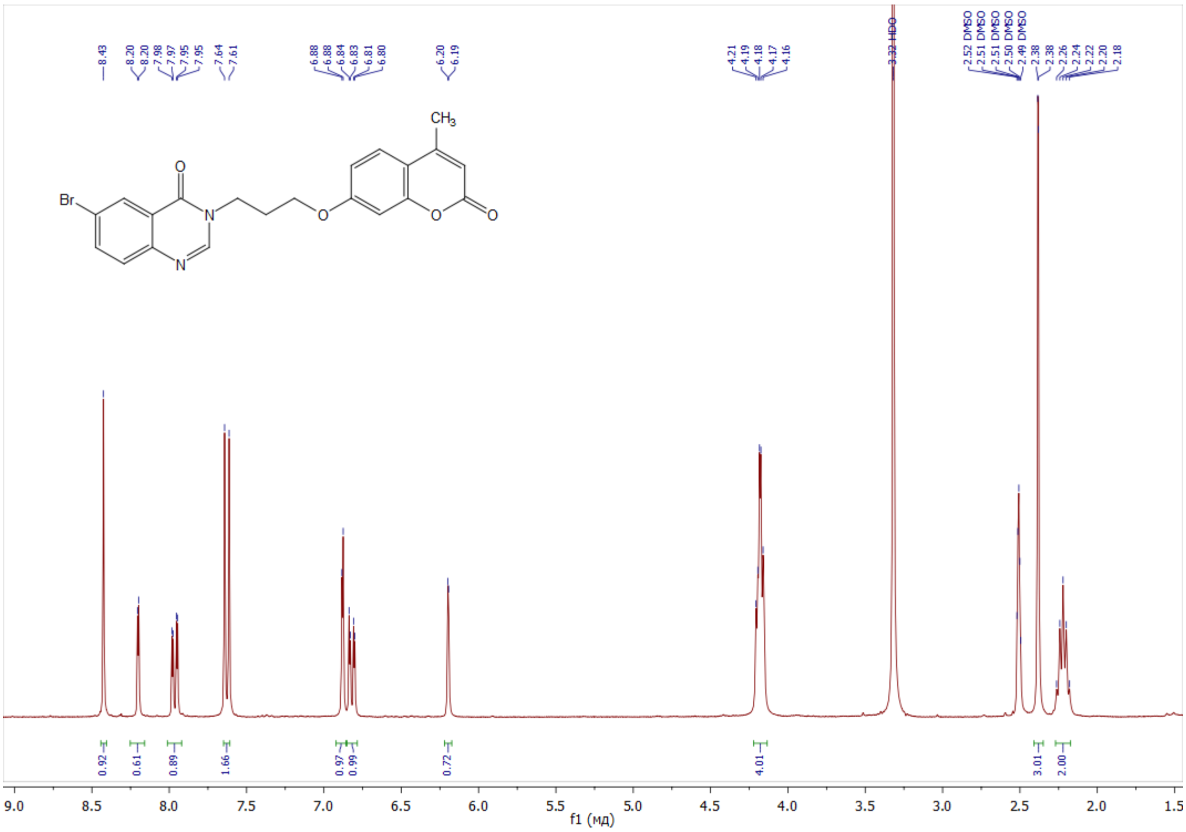

Figure S13 ^{1}H NMR spectrum of compound **3e** in DMSO-*d6*

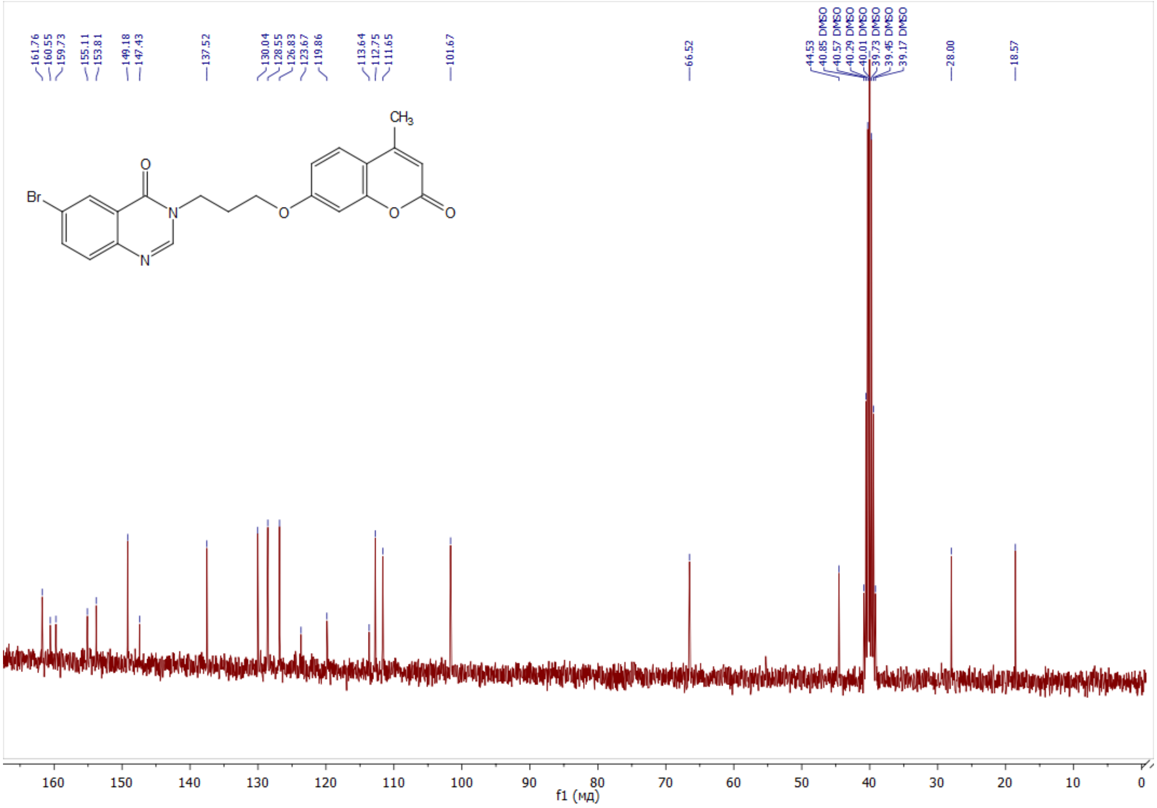

Figure S14 ^{13}C NMR spectrum of compound **3e** in DMSO-*d6*

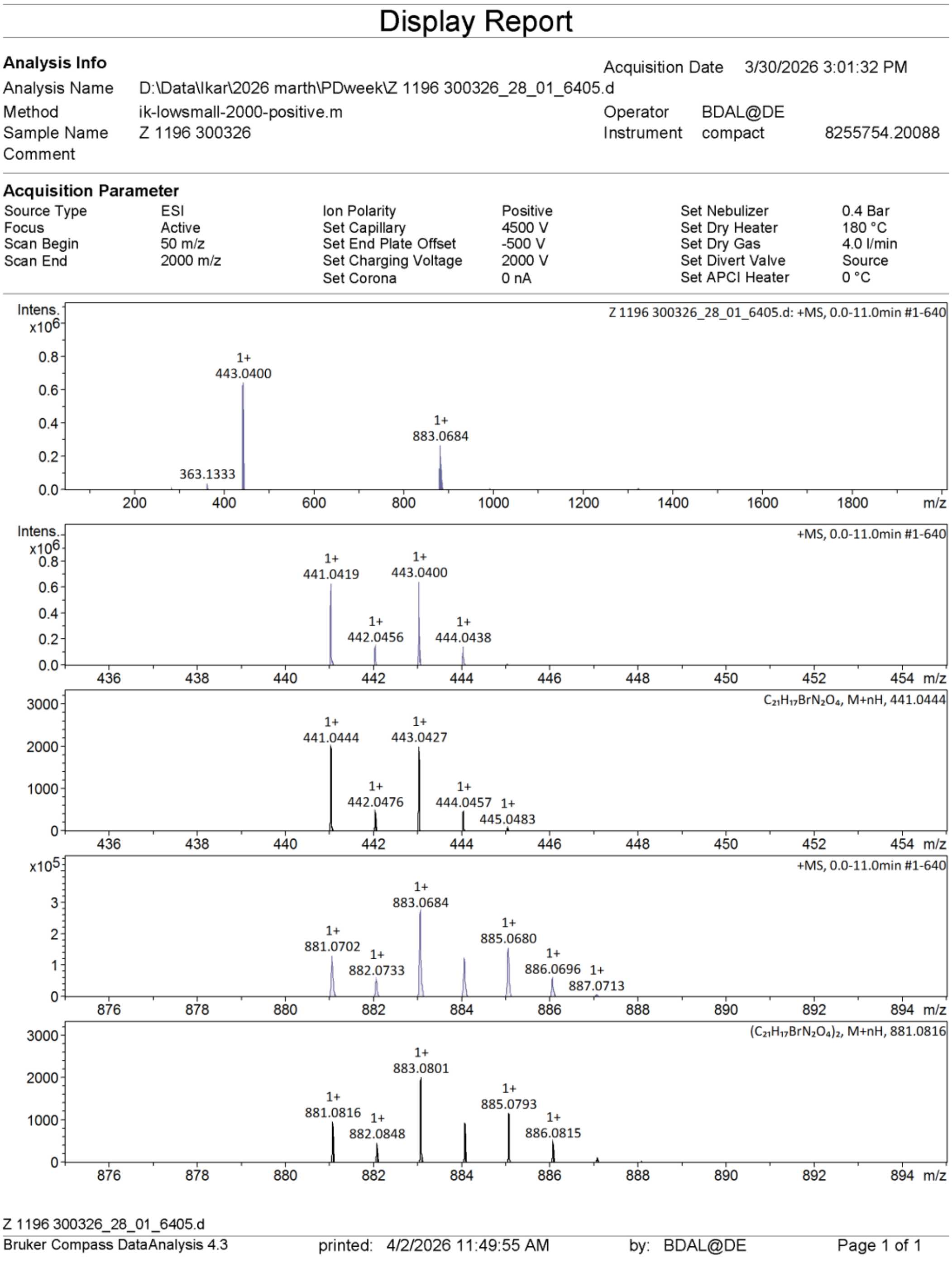

Figure S15: HRMS spectrum of compound **3e**

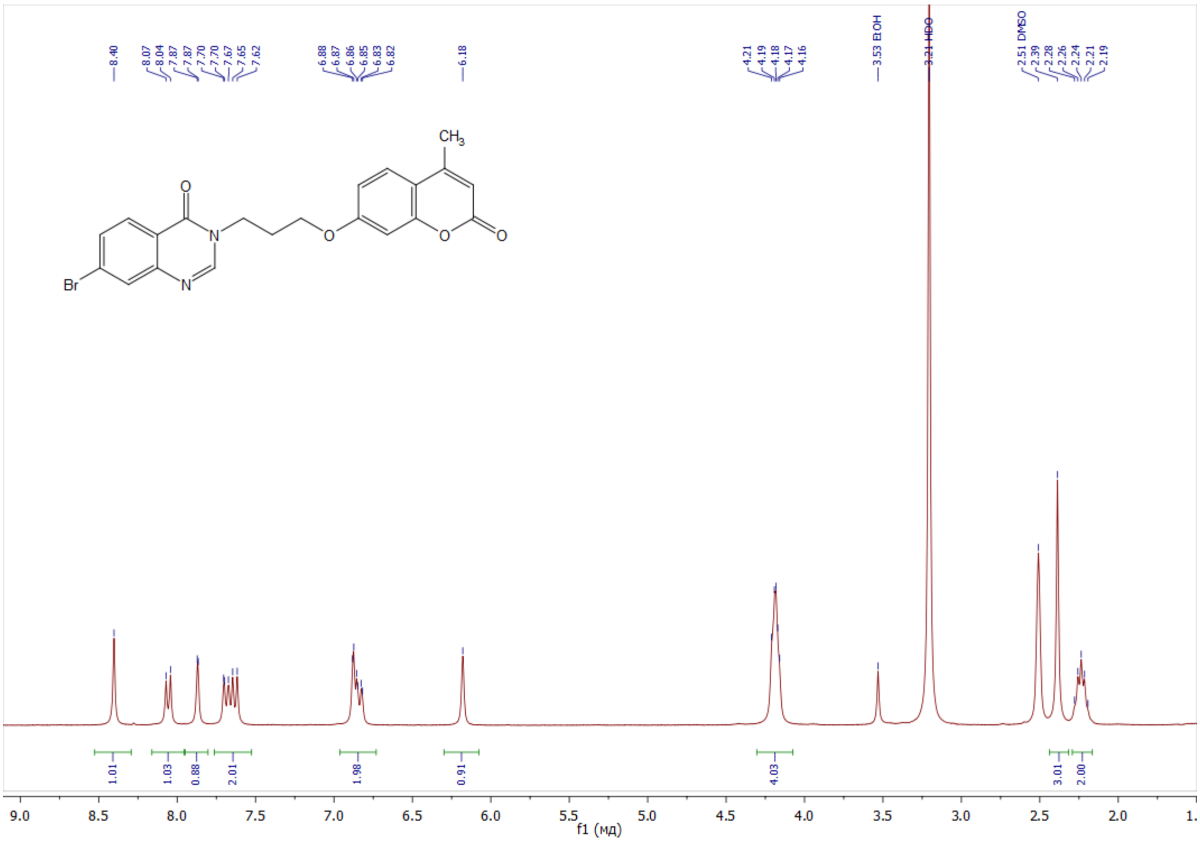

Figure S16 ^{1}H NMR spectrum of compound **3f** in DMSO-*d*6

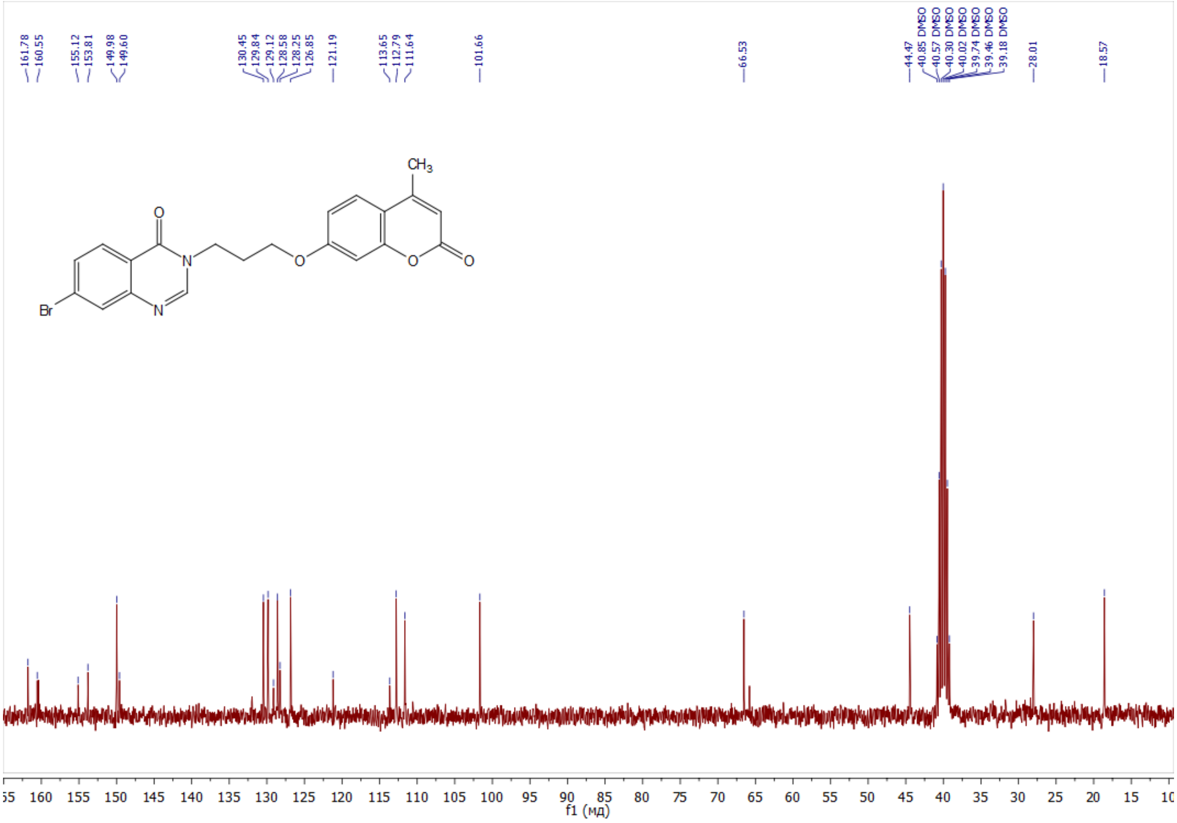

Figure S17 ^{13}C NMR spectrum of compound **3f** in DMSO-*d*6

Display Report

Analysis Info

Analysis Name	D:\Data\Ikar\2026 april\winterweek\Z 1197 300426_23_01_6624.d	Acquisition Date	4/30/2026 1:53:28 PM
Method	ik-lowsmall-2000-positive.m	Operator	BDAL@DE
Sample Name	Z 1197 300426	Instrument	compact 8255754.20088
Comment			

Acquisition Parameter

Source Type	ESI	Ion Polarity	Positive	Set Nebulizer	0.4 Bar
Focus	Active	Set Capillary	4500 V	Set Dry Heater	180 °C
Scan Begin	50 m/z	Set End Plate Offset	-500 V	Set Dry Gas	4.0 l/min
Scan End	2000 m/z	Set Charging Voltage	2000 V	Set Divert Valve	Source
		Set Corona	0 nA	Set APCI Heater	0 °C

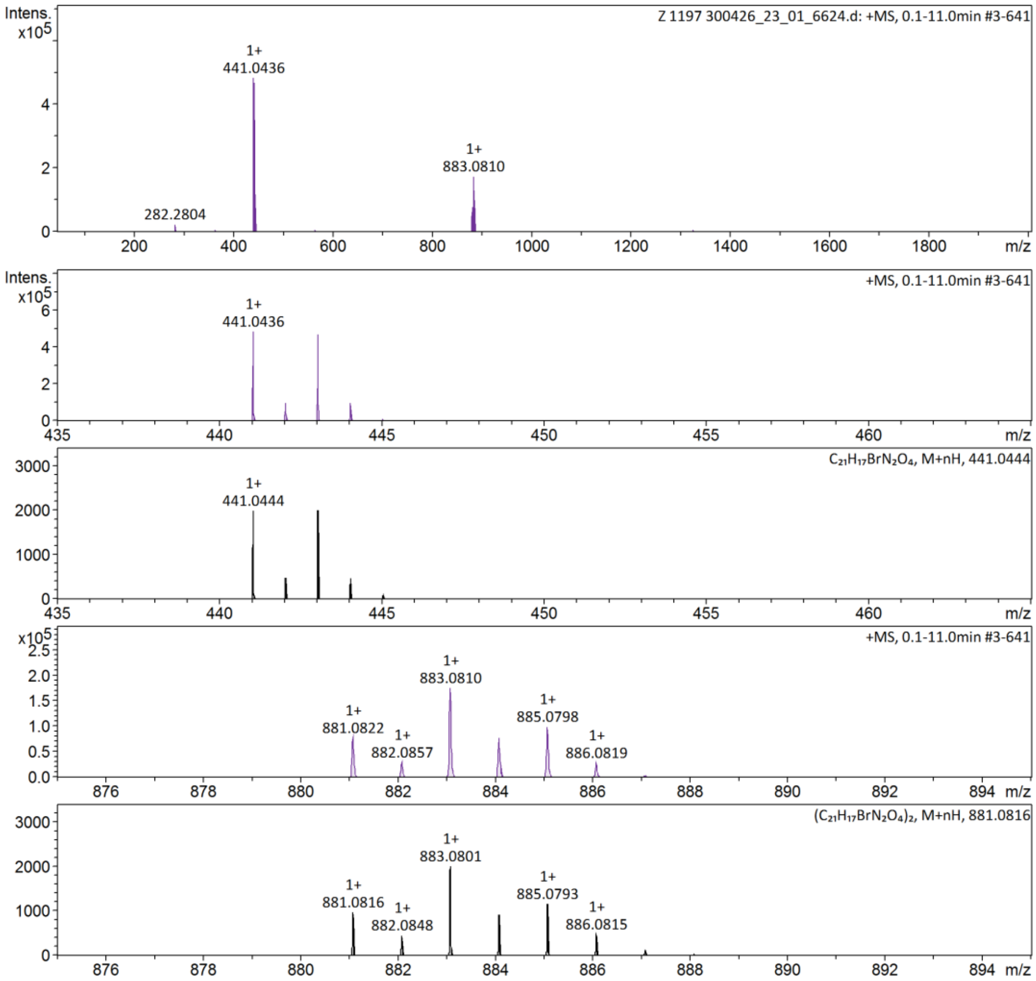

Z 1197 300426_23_01_6624.d

Bruker Compass DataAnalysis 4.3 printed: 4/30/2026 2:31:05 PM by: BDAL@DE Page 1 of 1

Figure S18: HRMS spectrum of compound **3f**

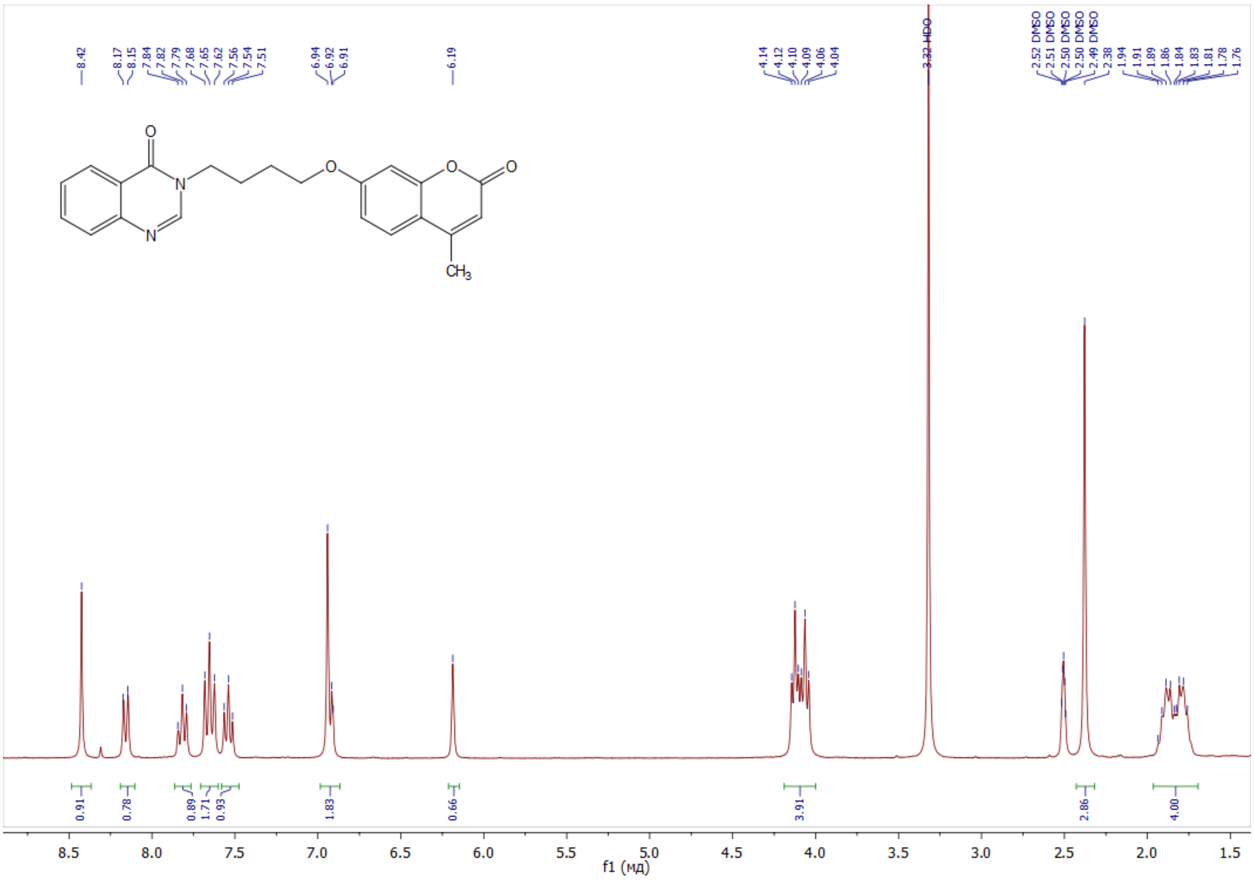

Figure S19 ^{1}H NMR spectrum of compound **3g** in DMSO-*d*6

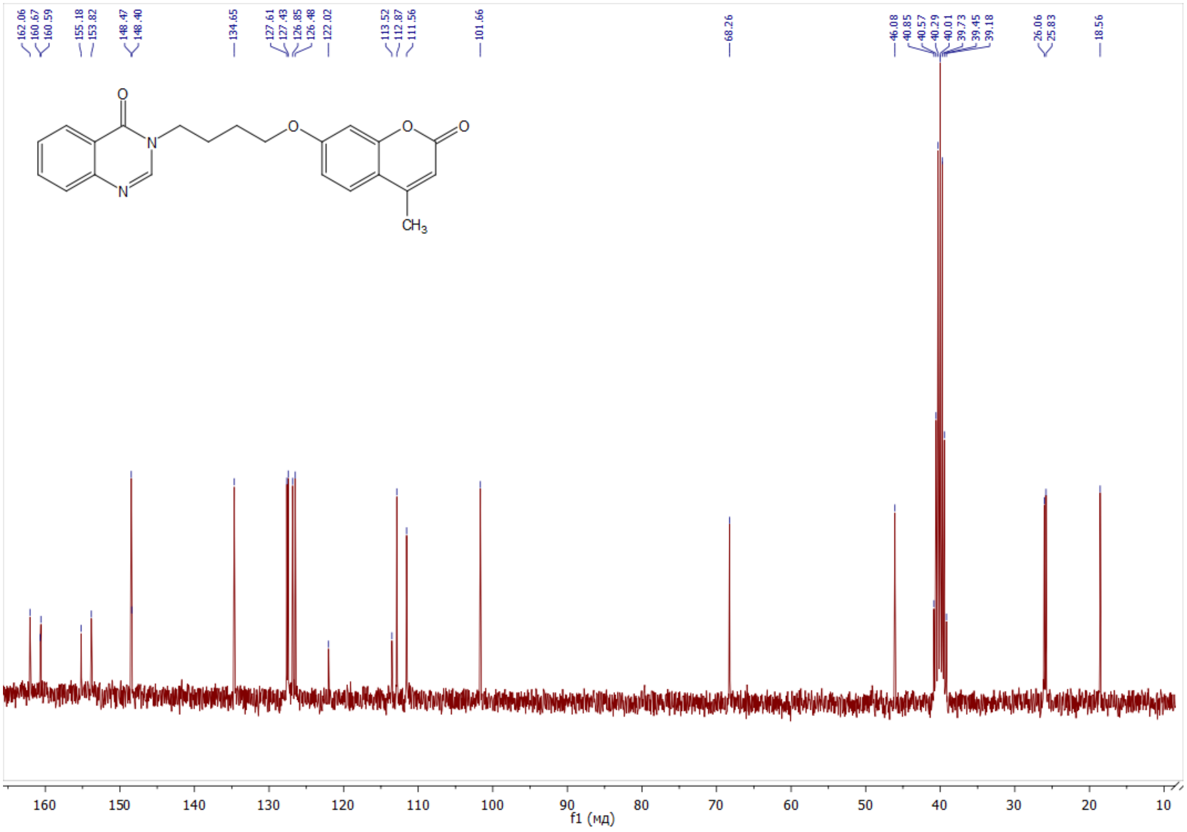

Figure S20 ^{13}C NMR NMR spectrum of compound **3g** in DMSO-*d*6

Display Report

Analysis Info

Analysis Name	D:\Data\Ikar\2026 marth\PDweek\Z 1190 300326_25_01_6402.d	Acquisition Date	3/30/2026 2:05:50 PM
Method	ik-lowsmall-2000-positive.m	Operator	BDAL@DE
Sample Name	Z 1190 300326	Instrument	compact 8255754.20088
Comment			

Acquisition Parameter

Source Type	ESI	Ion Polarity	Positive	Set Nebulizer	0.4 Bar
Focus	Active	Set Capillary	4500 V	Set Dry Heater	180 °C
Scan Begin	50 m/z	Set End Plate Offset	-500 V	Set Dry Gas	4.0 l/min
Scan End	2000 m/z	Set Charging Voltage	2000 V	Set Divert Valve	Source
		Set Corona	0 nA	Set APCI Heater	0 °C

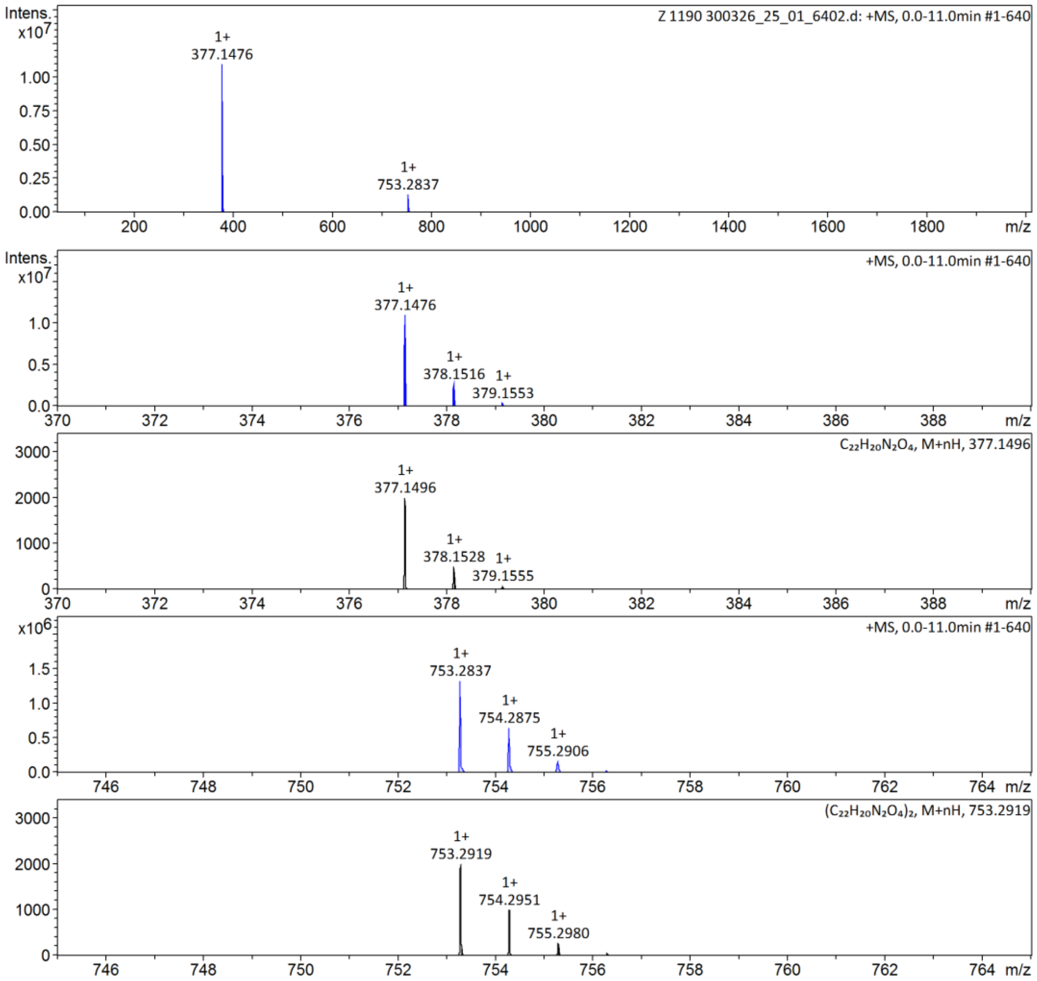

Z 1190 300326_25_01_6402.d

Bruker Compass DataAnalysis 4.3 printed: 4/2/2026 11:39:00 AM by: BDAL@DE Page 1 of 1

Figure S21: HRMS spectrum of compound **3g**

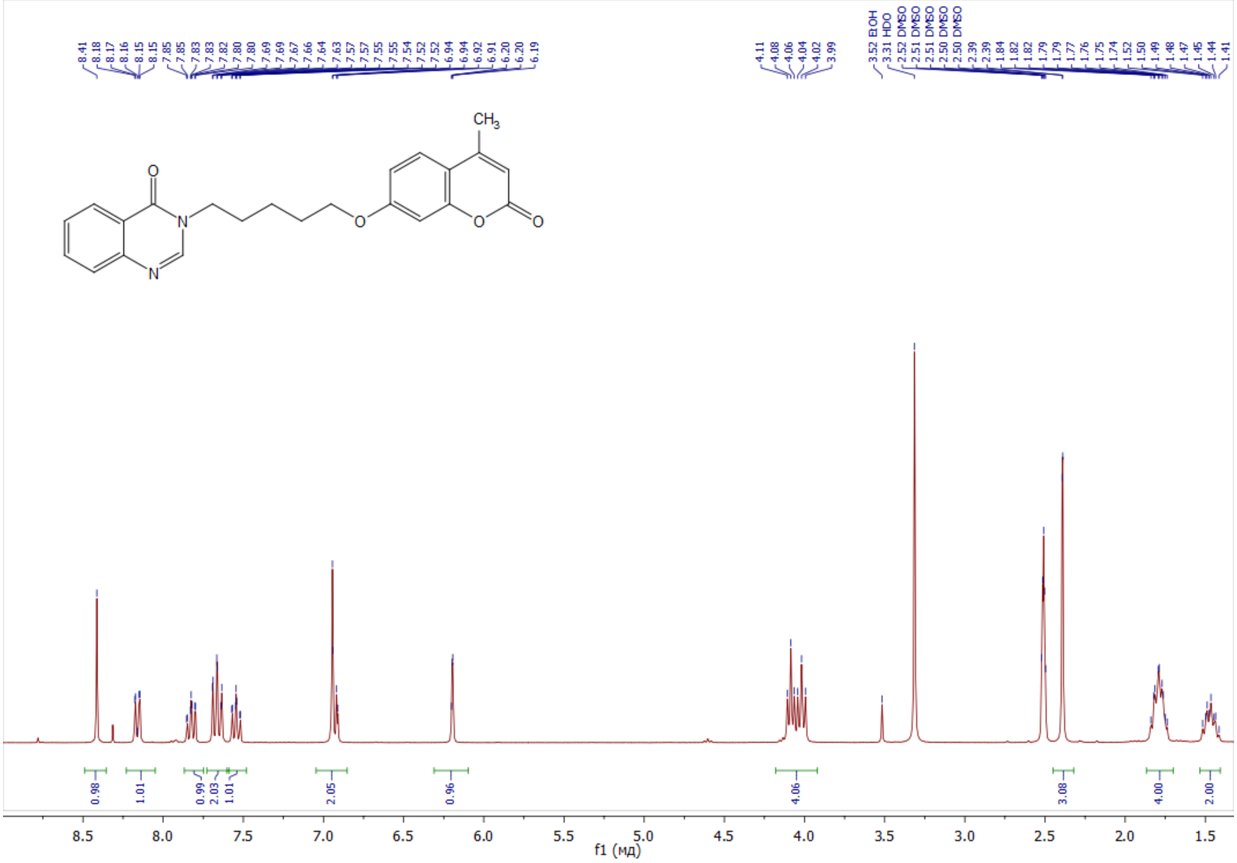

Figure S22 ^{1}H NMR spectrum of compound **3h** in DMSO-*d*6

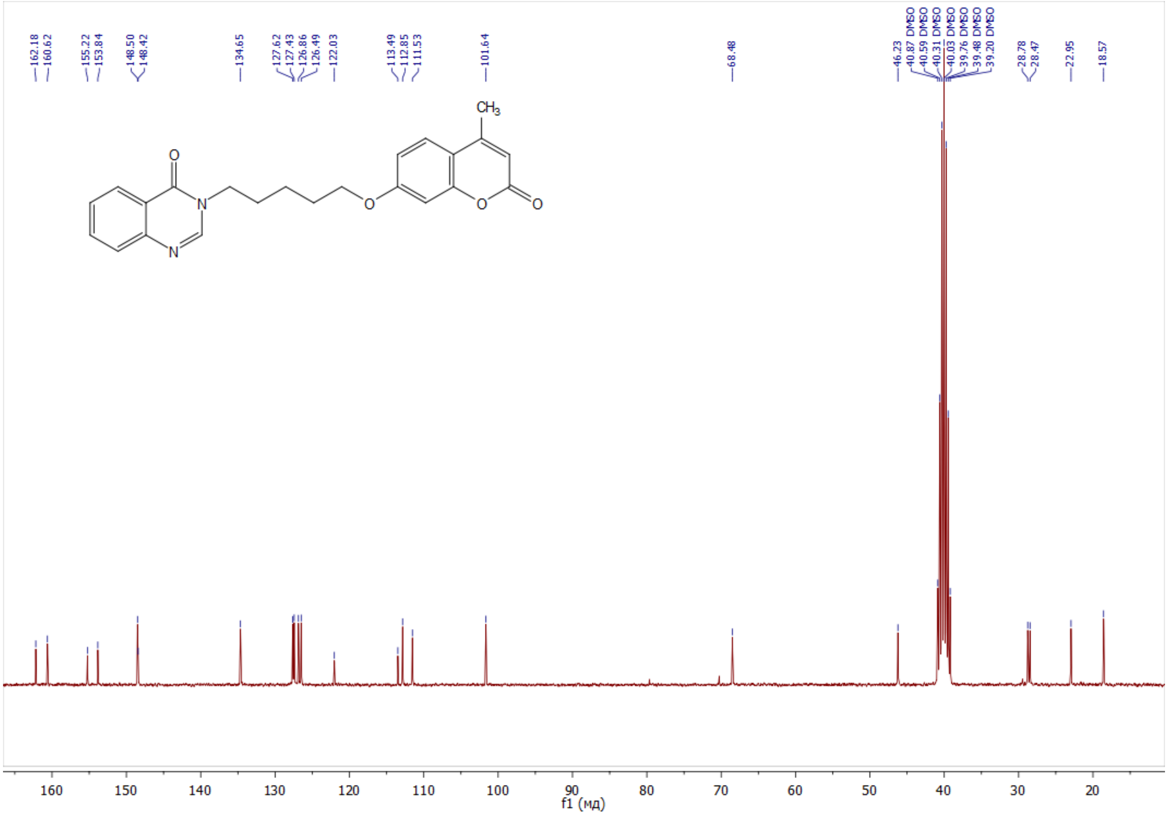

Figure S23 ^{13}C NMR NMR spectrum of compound **3h** in DMSO-*d*6

Display Report

Analysis Info

Analysis Name	D:\Data\Ikar\2026 marth\PDweek\Z 1159 p 310326_22_01_6424.d	Acquisition Date	3/31/2026 4:34:08 PM
Method	ik-lowsmall-2000-positive.m	Operator	BDAL@DE
Sample Name	Z 1159 p 310326	Instrument	compact 8255754.20088
Comment			

Acquisition Parameter

Source Type	ESI	Ion Polarity	Positive	Set Nebulizer	0.4 Bar
Focus	Active	Set Capillary	4500 V	Set Dry Heater	180 °C
Scan Begin	50 m/z	Set End Plate Offset	-500 V	Set Dry Gas	4.0 l/min
Scan End	2000 m/z	Set Charging Voltage	2000 V	Set Divert Valve	Source
		Set Corona	0 nA	Set APCI Heater	0 °C

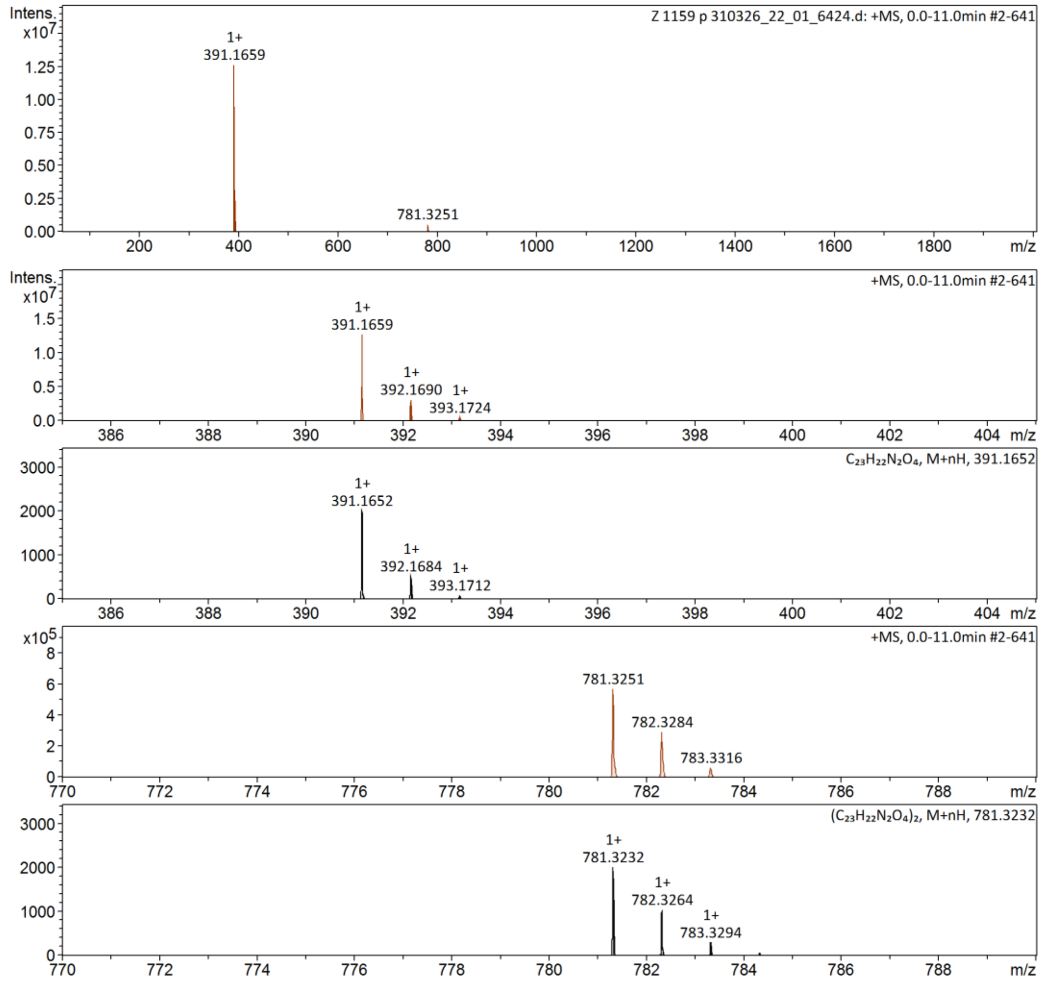

Z 1159 p 310326_22_01_6424.d

Bruker Compass DataAnalysis 4.3 printed: 4/2/2026 11:29:54 AM by: BDAL@DE Page 1 of 1

Figure S24: HRMS spectrum of compound **3h**

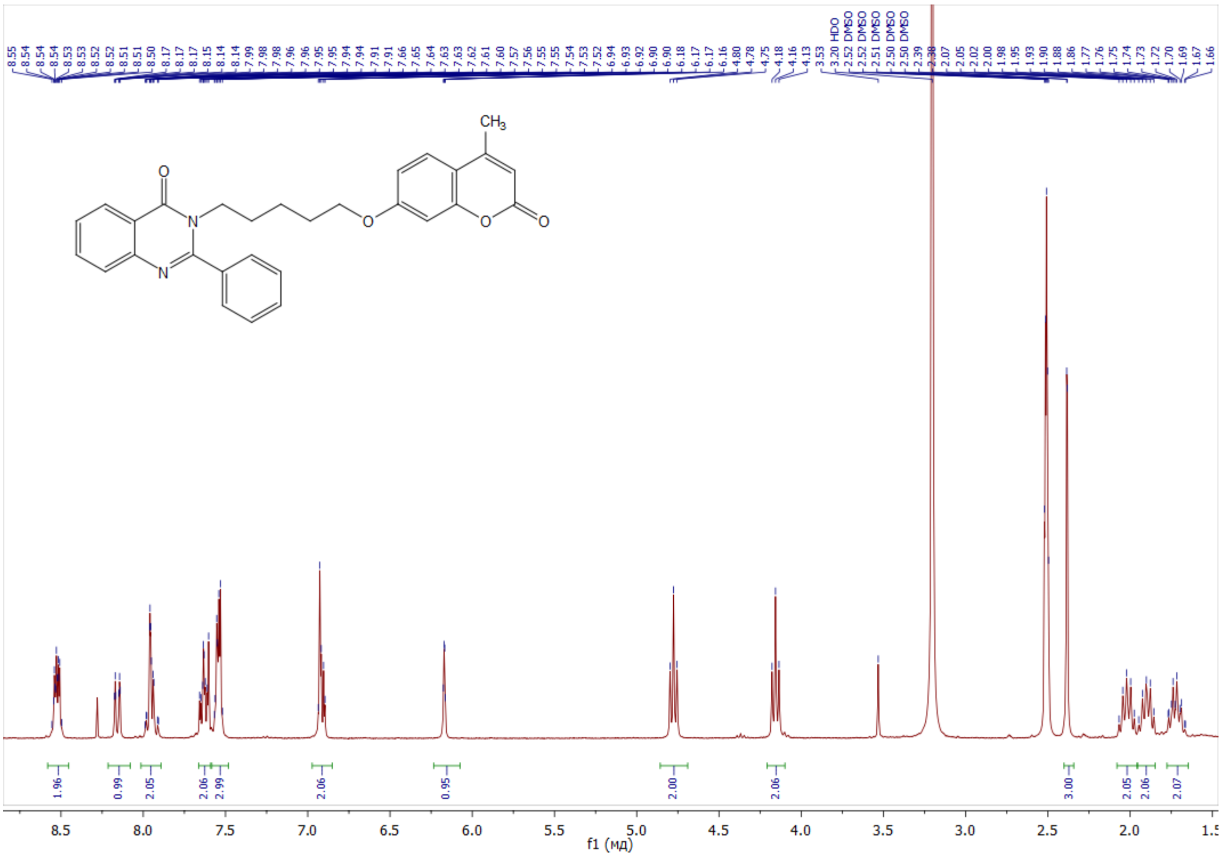

Figure S25 ^{1}H NMR spectrum of compound **3i** in DMSO-*d*6

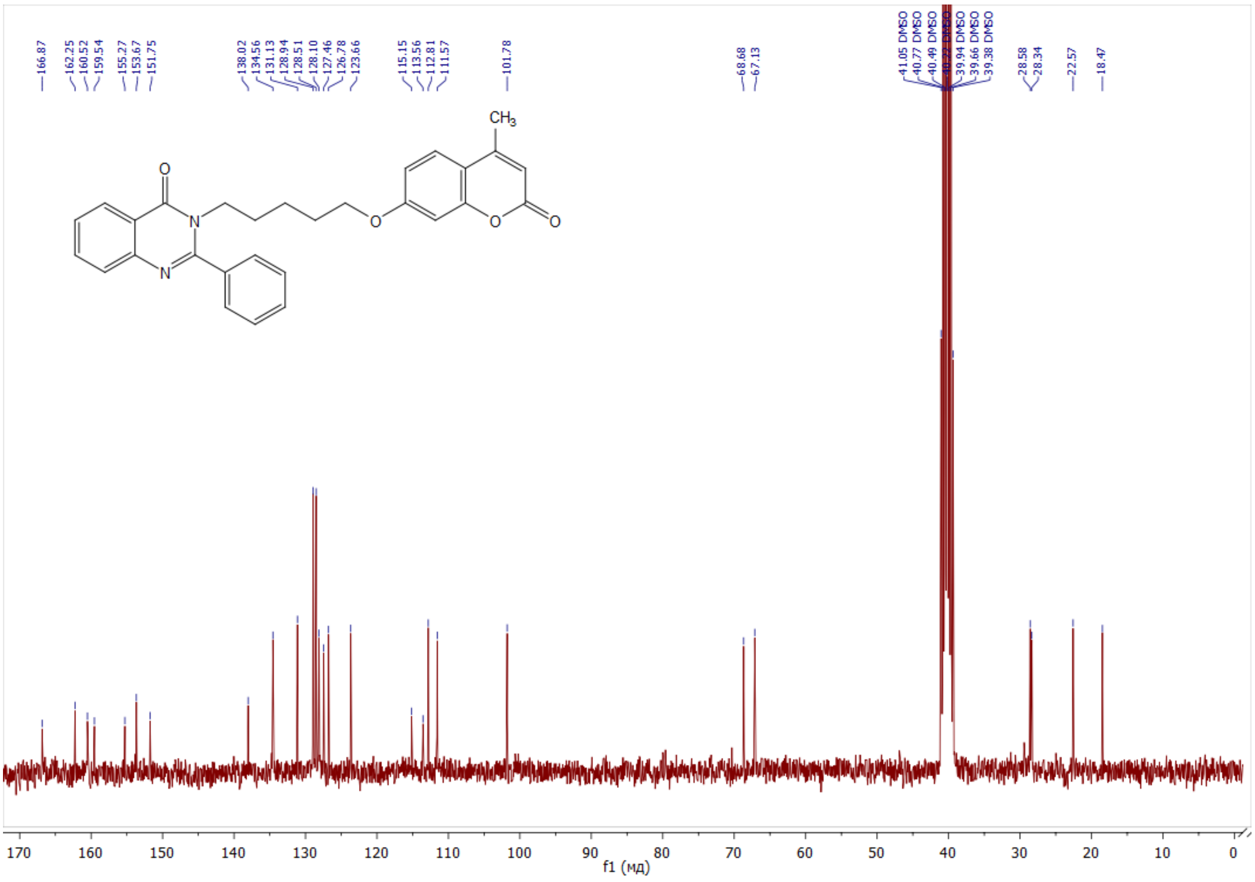

Figure S26 ^{13}C NMR spectrum of compound **3i** in DMSO-*d*6

Display Report

Analysis Info

Analysis Name	D:\Data\Ikar\2026 marth\PDweek\Z 1160 300326_24_01_6401.d	Acquisition Date	3/30/2026 1:47:17 PM
Method	ik-lowsmall-2000-positive.m	Operator	BDAL@DE
Sample Name	Z 1160 300326	Instrument	compact 8255754.20088
Comment			

Acquisition Parameter

Source Type	ESI	Ion Polarity	Positive	Set Nebulizer	0.4 Bar
Focus	Active	Set Capillary	4500 V	Set Dry Heater	180 °C
Scan Begin	50 m/z	Set End Plate Offset	-500 V	Set Dry Gas	4.0 l/min
Scan End	2000 m/z	Set Charging Voltage	2000 V	Set Divert Valve	Source
		Set Corona	0 nA	Set APCI Heater	0 °C

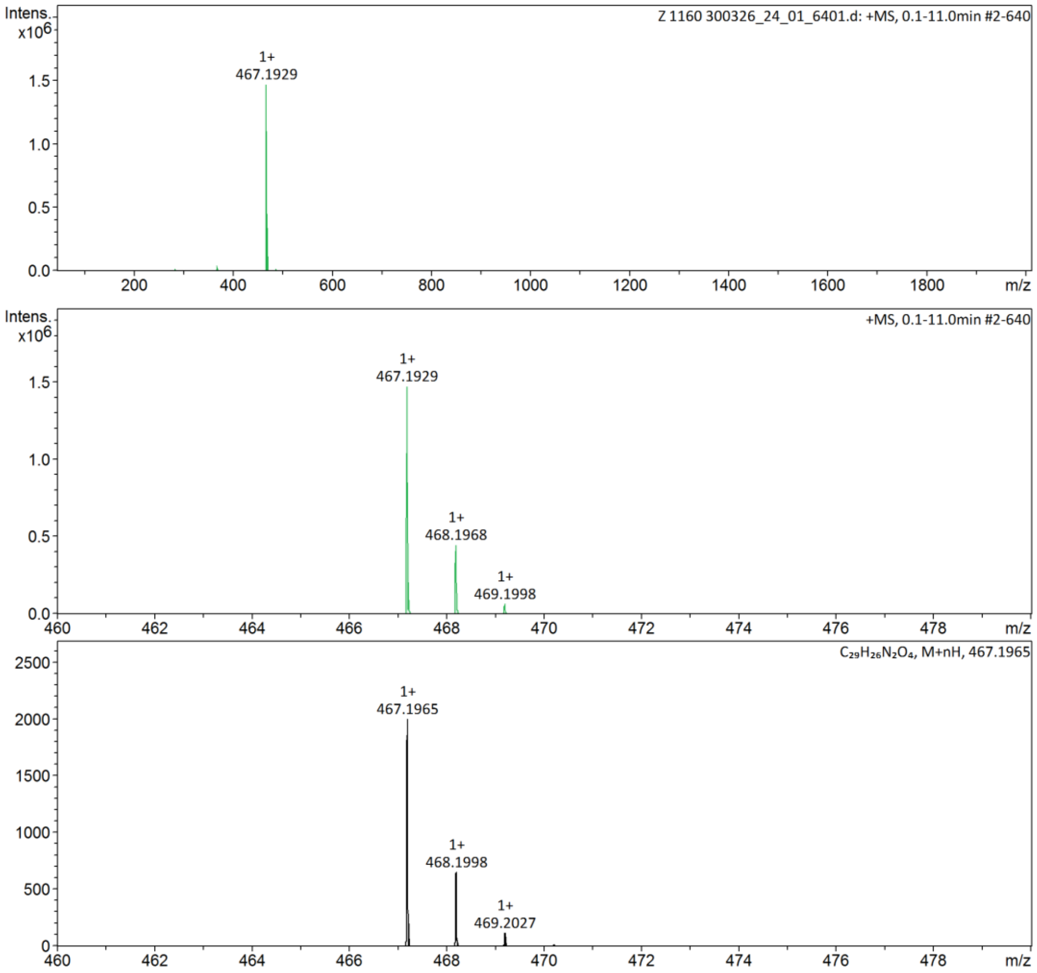

Z 1160 300326_24_01_6401.d

Bruker Compass DataAnalysis 4.3 printed: 4/2/2026 11:34:10 AM by: BDAL@DE Page 1 of 1

Figure S27: HRMS spectrum of compound **3i**

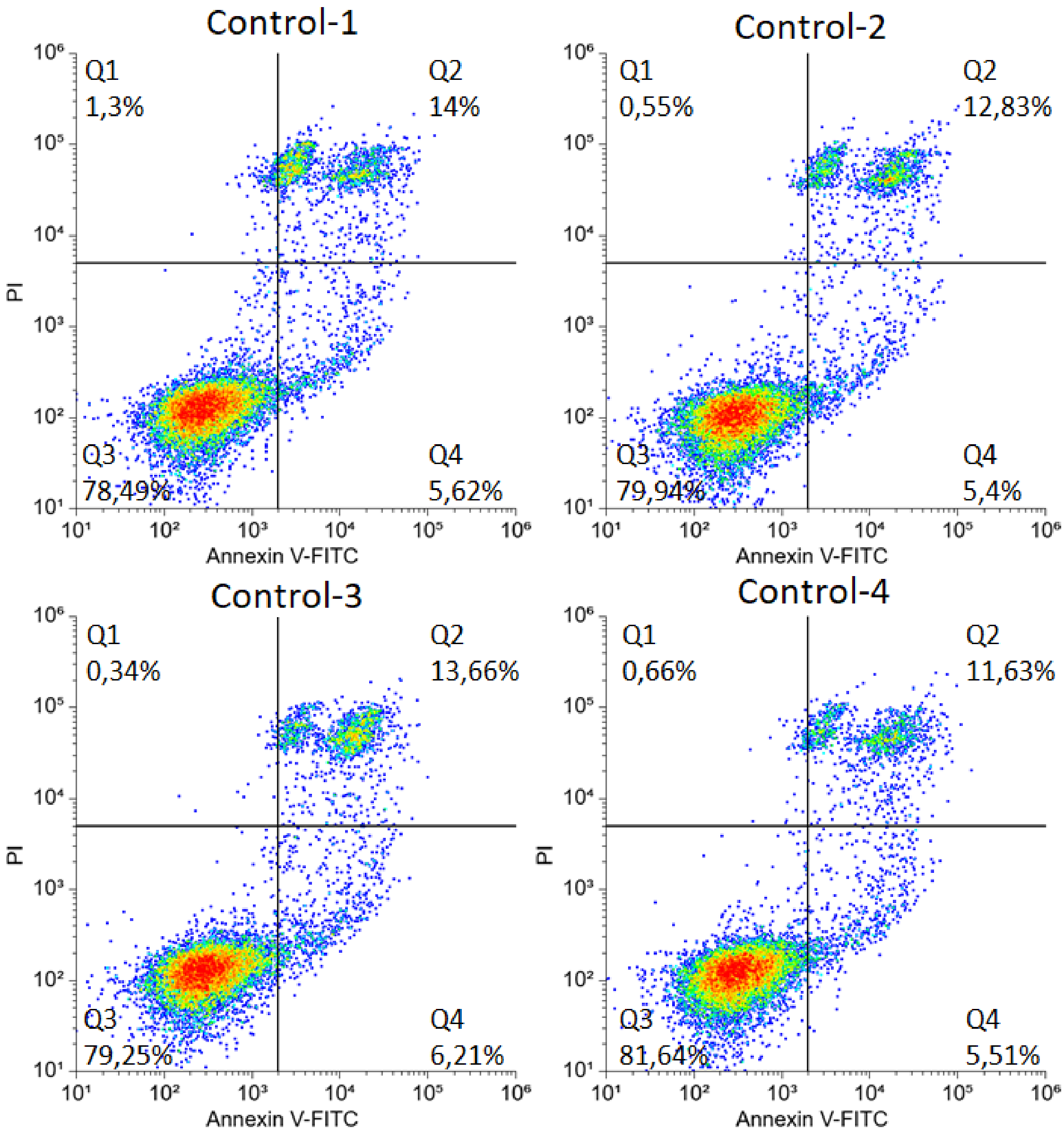

Figure S28. Dot plots of Annexin V-FITC and PI stained control Jurkat cells.

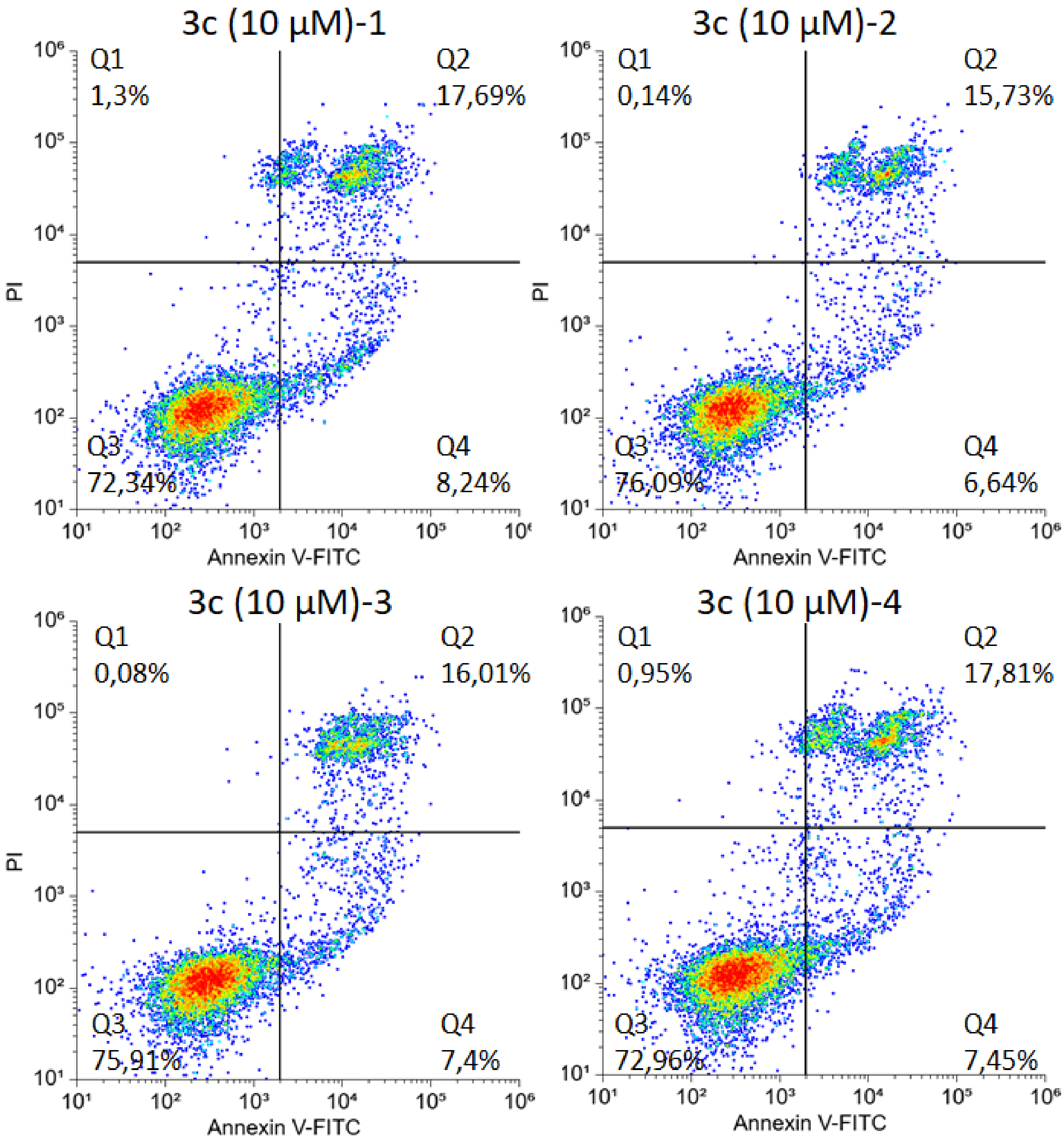

Figure S29. Dot plots of Annexin V-FITC and PI stained Jurkat cells for evaluation of apoptosis induced by 10 µM of **3c**.

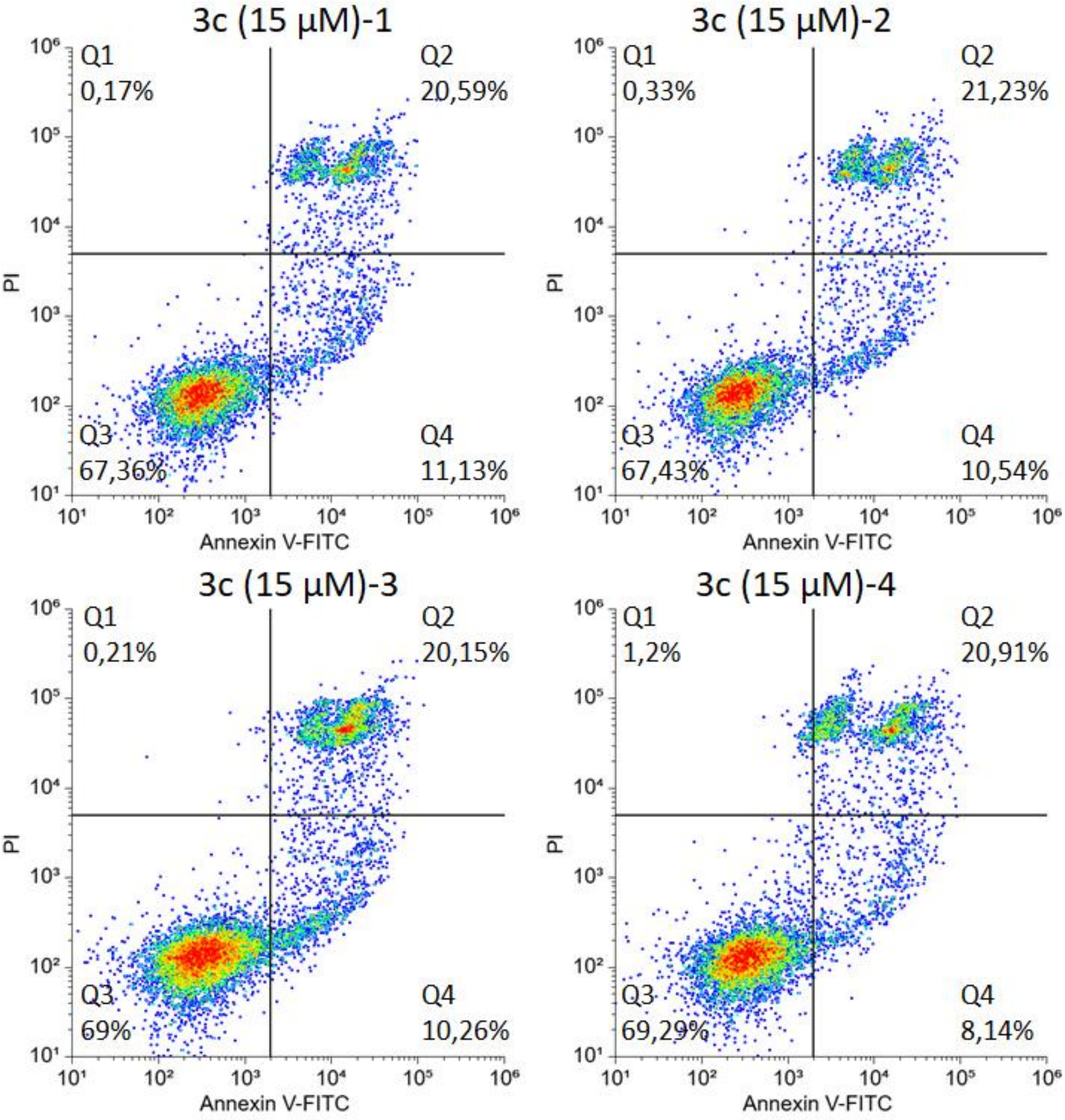

Figure S30. Dot plots of Annexin V-FITC and PI stained Jurkat cells for evaluation of apoptosis induced by 15 µM of **3c**.

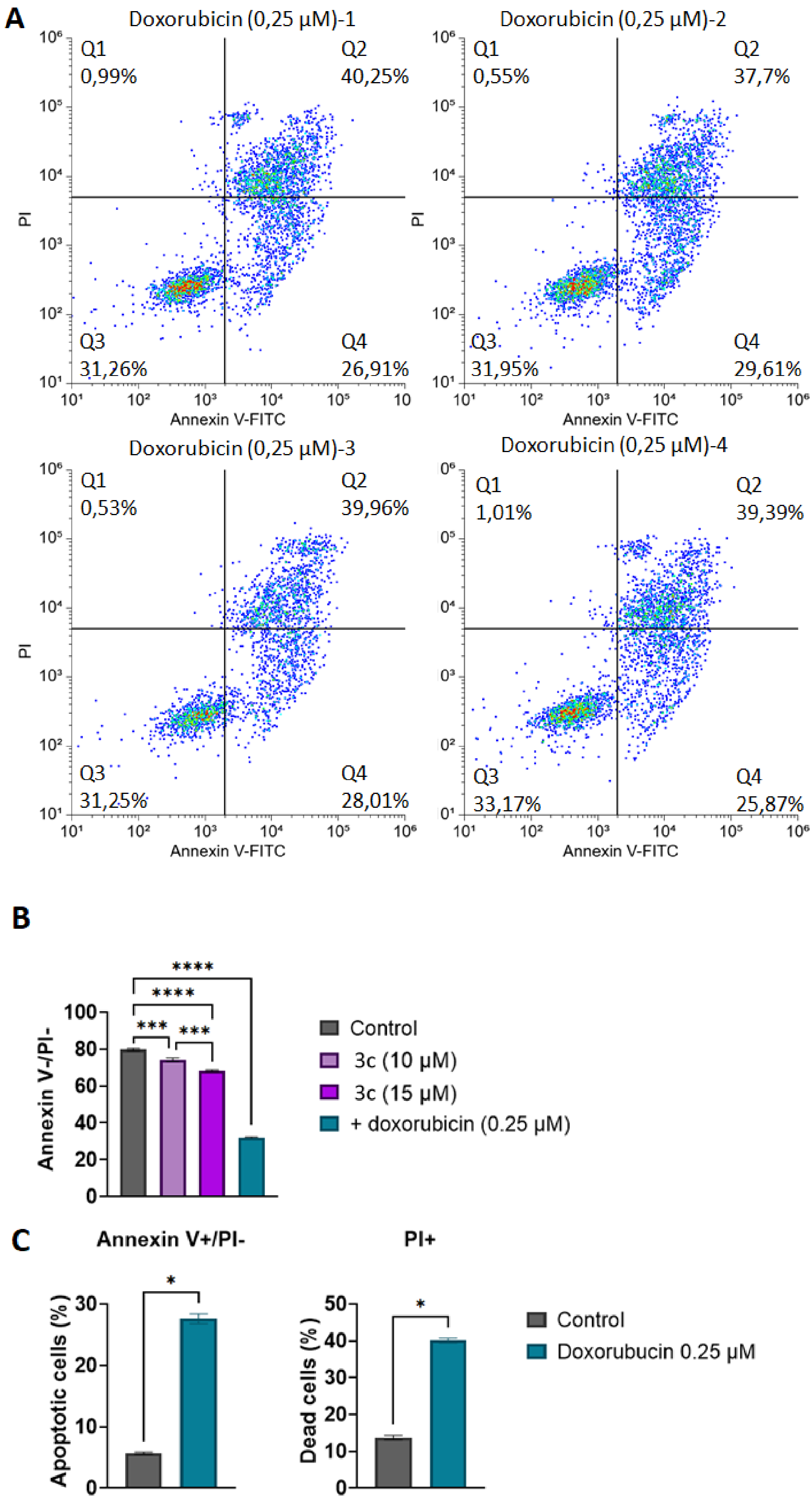
A
Doxorubicin (0,25 µM)-1
Q1 0,99%
Q2 40,25%
Q3 31,26%
Q4 26,91%
Doxorubicin (0,25 µM)-2
Q1 0,55%
Q2 37,7%
Q3 31,95%
Q4 29,61%
Doxorubicin (0,25 µM)-3
Q1 0,53%
Q2 39,96%
Q3 31,25%
Q4 28,01%
Doxorubicin (0,25 µM)-4
Q1 1,01%
Q2 39,39%
Q3 33,17%
Q4 25,87%
PI
Annexin V-FITC
B
Annexin V-/PI-
Control
3c (10 µM)
3c (15 µM)
+ doxorubicin (0.25 µM)
C
Annexin V+/PI-
PI+
Apoptotic cells (%)
Dead cells (%)
Control
Doxorubucin 0.25 µM

Figure S31. Dot plots of Annexin V-FITC and PI stained Jurkat cells for evaluation of apoptosis induced by 0.25 μM of doxorubicin (A), procents of annexin V-/PI- Jurkat cells (with intact membranes) (B) and procents of apoptotic and dead Jurkat cells after treatment with 0,25 μM of doxorubicin (C). Data are presented as mean ± SEM, n=4. Adjusted p-values: *$p \leq 0.05$, ***$p \leq 0.001$, ****$p \leq 0.0001$, based on a one-way analysis of variance (ANOVA) with Tukey's post hoc test (B) or Mann-Whitney nonparametric test (C).
